# Role of formaldehyde in promoting aromatic selectivity during methanol conversion over gallium-modified zeolites

**DOI:** 10.1038/s42004-022-00771-8

**Published:** 2022-11-19

**Authors:** Wu Wen, Tianci Xiao, Beibei Feng, Chaoqun Zhou, Jian Li, Hao Ma, Zhongyue Zhou, Ying Zhang, Jiuzhong Yang, Zhandong Wang, Fei Qi, Jun Bao, Chengyuan Liu, Yang Pan

**Affiliations:** 1 National Synchrotron Radiation Laboratory, University of Science and Technology of China, Hefei, 230029 Anhui P. R. China; 2 Key Laboratory for Power Machinery and Engineering of Ministry of Education, Shanghai Jiao Tong University, Shanghai, 200240 P. R. China; 3 Shanghai Research Institute of Petrochemical Technology SINOPEC, Shanghai, 201208 P. R. China; 4 Department of Chemistry, University of Science and Technology of China, Hefei, 230029 Anhui P. R. China

**Keywords:** Catalytic mechanisms, Heterogeneous catalysis, Porous materials

## Abstract

Gallium-modified HZSM-5 zeolites are known to increase aromatic selectivity in methanol conversion. However, there are still disputes about the exact active sites and the aromatic formation mechanisms over Ga-modified zeolites. In this work, in situ synchrotron radiation photoionization mass spectrometry (SR-PIMS) experiments were carried out to study the behaviors of intermediates and products during methanol conversion over Ga-modified HZSM-5. The increased formaldehyde (HCHO) yield over Ga-modified HZSM-5 was found to play a key role in the increase in aromatic yields. More HCHO was deemed to be generated from the direct dehydrogenation of methanol, and Ga_2_O_3_ in Ga-modified HZSM-5 was found to be the active phase. The larger increase in aromatic production over Ga-modified HZSM-5 after reduction‒oxidation treatment was found to be the result of redispersed Ga_2_O_3_ with smaller size generating a larger amount of HCHO. This study provides some new insights into the internal driving force for promoting the production of aromatics over Ga-modified HZSM-5.

## Abstract (web summary)

Gallium-modified HZSM-5 zeolites demonstrate increased selectivity towards aromatics during methanol conversion, but the mechanism and the nature of the active sites that lead to this enhanced selectivity are poorly understood. Here, in situ synchrotron radiation photoionization mass spectrometry experiments reveal that increased formaldehyde generation over Ga_2_O_3_ species plays a key role in promoting the production of aromatic compounds.

## Introduction

The catalytic conversion of methanol-to-hydrocarbons (MTH) is considered to be a promising route, which can convert coal and natural gas to more valuable fuels and commodity chemicals through methanol (MeOH)^1–3^. Aromatic production through catalytic conversion of MeOH is a highly attractive alternative to the process of producing aromatics from petroleum resources. It is well known that metal-modified ZSM-5 zeolites have distinct performance for increasing aromatic selectivity in MeOH conversion^4–9^. For Ga-modified HZSM-5 zeolites, both cationic metal species (Ga^3+^) and/or metal oxides (Ga_2_O_3_) have been reported to result in a significant enhancement in aromatic production^7,10–13^. In Ga-modified HZSM-5 prepared by impregnation or ion exchange, previous works suggested that the substitution of Brønsted acid sites (BASs) by cationic Ga species can bring about strong Lewis acid sites (LASs), and the synergistic effect derived from the close spatial proximity between cationic Ga species and BASs leads to an enhanced Brønsted acidity^7^. The cationic Ga species cooperate with BASs to facilitate the dehydrogenation-aromatization processes of cycloalkenes, which can be called the LAS-induced aromatic formation pathway^12^. Physical mixtures of Ga_2_O_3_ and HZSM-5 can also improve the selectivity of aromatics^5,10,11^. It suggested that there may exist some certain active sites located in the interface region between Ga_2_O_3_ and zeolite to promote MeOH into aromatics^11^.

Formaldehyde (HCHO) is an important active intermediate in the MTH reaction, which has aroused wide interest from many investigators recently^14–17^. HCHO is considered to be involved in aromatic formation in the MTH reaction, i.e., the HCHO-induced aromatic formation pathway^18,19^. Lercher et al. suggested that HCHO can react with olefins to form dienes via Prins reaction (HCHO + olefins → dienes + H_2_O), and dienes react stepwise with HCHO to form H-poor products, aromatics and eventually cokes^14^. The researchers studied the behavior of HCHO in MTH by adding HCHO into the reactants, and found that the addition of HCHO can significantly increase the selectivity to aromatics^15,20,21^, and accelerate catalyst deactivation^22,23^. In our previous work, the critical role of HCHO in the mechanisms of aromatic formation was confirmed with the excellent time-resolved profiles obtained by in situ synchrotron radiation photoionization mass spectrometry (SR-PIMS)^24^.

Although the importance of HCHO in the MTH reaction is well established, the exact active centers on Ga-modified HZSM-5 zeolites and how they affect the formation of HCHO under MTH conditions are still not clear. In this work, questions like how HCHO was generated on the Ga-modified HZSM-5 and what the active sites were, were studied systematically. The new insights into the HCHO-induced aromatic formation pathway due to the introduction of Ga species supplemented the previous dehydrogenation-aromatization mechanism.

## Results and discussion

### Formaldehyde trend related to aromatics

In this work, in situ SR-PIMS (Supplementary Fig. 1) was further utilized to explore the formation and function of HCHO in MTH over Ga-modified HZSM-5^24^. To alleviate the secondary reactions of HCHO, a 2 Torr pressure was applied to the catalytic reactor. The Ga-modified HZSM-5 was prepared by impregnation and ion exchange methods, which are denoted as Ga(IM)HZSM-5 and Ga(IE)HZSM-5. Ga(IM-A/B/C)HZSM-5 represents a Ga(IM)HZSM-5 with different Ga loadings (Table 1).

**Table 1:** Structural and acidic properties of the parent HZSM-5 and Ga-modified HZSM-5.

Sample	Ga loading^a^ (wt%)	Ga/Al ratio^a^	Si/Ga ratio^b^	Relative crystallinity^c^ (%)	S_BET_ (m^2^g^−1^)	V_micro_ (cm^3^g^−1^)	Acid amount^d^ (μmol g^−1^)			Total	Acid sites^e^ (μmol g^−1^)		
							weak	medium	strong		BAS	LAS	B/L
HZSM-5	0	–	–	100.0	305	0.11	273.1	196.2	168.2	637.5	334.9	86.4	3.9
Ga(IM-A)HZSM-5	0.98	0.6	11.8	99.0	289	0.11	260.5	193.8	154.5	608.8	308.8	90.7	3.4
Ga(IM-B)HZSM-5	2.4	1.4	6.8	99.6	272	0.11	250.1	182.5	143.2	575.8	270.6	105.5	2.6
Ga(IM-B)HZSM-5(redox)	2.5	1.4	26.1	98.8	271	0.1	262.6	199.8	120.4	582.9	100.9	162.7	0.6
Ga(IM-C)HZSM-5	4.6	2.7	5.6	95.5	265	0.1	247.8	183.7	143.3	574.8	266.2	110.1	2.4
Ga(IE)HZSM-5	0.2	0.1	90.2	104.0	264	0.1	232.4	180.8	139.6	552.8	183.0	168.4	0.8

^a^Determined by ICP-AES.

^b^Measured by XPS.

^c^Calculated from XRD.

^d^Measured by NH_3_-TPD.

^e^Measured by TF-IR of pyridine adsorption.

**Fig. 1:**
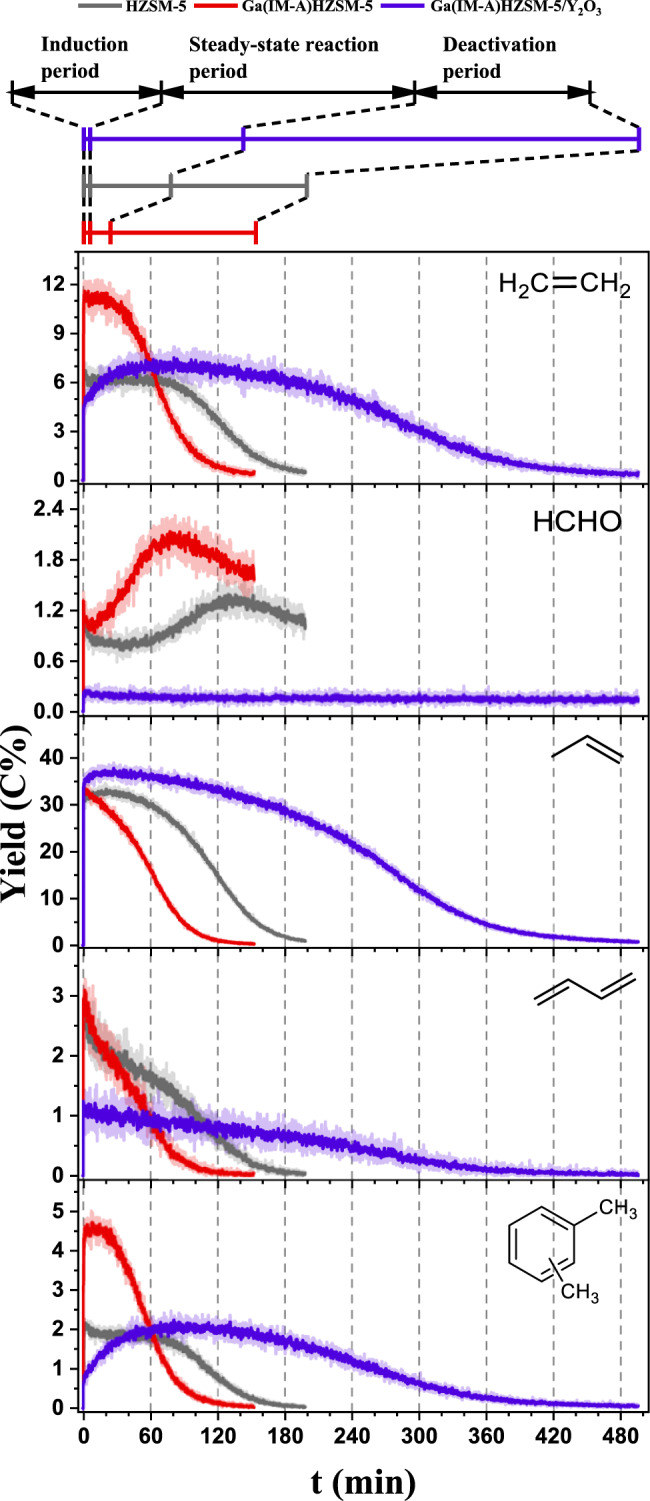
Product distribution after boosting or eliminating in formaldehyde. The real-time yields (C%) of C_2_H_4_, HCHO, propylene, 1,3-butadiene and C_8_ aromatics in MTH reaction over parent HZSM-5, Ga(IM-A)HZSM-5 and Ga(IM-A)HZSM-5/Y_2_O_3_. Reaction conditions: 400 °C; MeOH weight hourly space velocity (WHSV) was 12.52 g_MeOH_g_catalyst_^−1^h^−1^; pressure was 2 Torr; and each reaction proceeded until the C_2_H_4_ yield dropped to 0.5 C%. The original yield curves (transparent solid lines) have been smoothed as bright solid lines.

The time-resolved profiles of the main product yields in MTH over the parent HZSM-5 and Ga(IM-A)HZSM-5 at 400 °C are demonstrated in Fig. 1, where the induction, steady-state reaction, and deactivation periods could be distinguished. In terms of steady-state reaction period, compared with the parent HZSM-5, Ga(IM-A)HZSM-5 could produce more aromatics (including C_7_-C_9_ aromatics) (Fig. 1 and Supplementary Fig. 4). However, the propylene yield did not change much, while the yields of C_4_^=^ and C_5_^=^ olefins decreased slightly. Because the formation of ethylene is closely associated with the aromatic-based cycle in the dual-cycle mechanism of the MTH reaction, the yield of ethylene over Ga(IM-A)HZSM-5 increased obviously with the enhanced production of aromatics. Here, it is worth noting that the HCHO yield over Ga(IM-A)HZSM-5 was higher than that over HZSM-5, especially in the deactivation period. This promoting effect on aromatic formation was similar to the co-feeding of formaldehyde with methanol at the expense of catalyst lifetime due to severe coke deposition (Supplementary Fig. 5).

Y_2_O_3_ can decompose HCHO into CO and H_2_, and has little activity for the conversion of MeOH and other stable products in the MTH reaction^25^. Hence, the physical mixture of Ga(IM-A)HZSM-5 with Y_2_O_3_ (Ga(IM-A)HZSM-5/Y_2_O_3_) was introduced to remove HCHO in MTH and verify whether the increase in aromatic yields promoted by Ga(IM-A)HZSM-5 was tightly connected with the increased HCHO yield. As shown in Fig. 1, the HCHO yield over Ga(IM-A)HZSM-5/Y_2_O_3_ was significantly reduced in the whole reaction process, and the yields of aromatics over Ga(IM-A)HZSM-5/Y_2_O_3_ decreased to the level of the parent HZSM-5 in the steady-state reaction period. The yield of 1,3-butadiene derived from the Prins reaction also decreased. In addition, the variation in stable products with and without Y_2_O_3_ obtained by GC‒MS experiments under atmospheric pressure was similar to that of SR-PIMS experiments (Supplementary Table 2). These results indicated that the HCHO-induced aromatic formation pathway strengthened by Ga(IM)HZSM-5 played an important role in increasing aromatic production.

### Formaldehyde formation pathway over Ga-modified HZSM-5

After that, we need to further explore the formation mechanism of the more HCHO generated from Ga(IM)HZSM-5. HCHO in the MTH reaction was suggested to be mainly produced through three pathways, including disproportionation of MeOH on the acid sites of HZSM-5 (Eq. (1))^26–28^, hydrogen transfer from MeOH to olefins on LAS (Eq. (2))^14^ and catalytic dehydrogenation of MeOH (Eq. (3))^17^.

1 $$2{{{{{{\rm{CH}}}}}}}_{3}{{{{{\rm{OH}}}}}}\to {{{{{\rm{HCHO}}}}}}+{{{{{{\rm{CH}}}}}}}_{4}+{{{{{{\rm{H}}}}}}}_{2}{{{{{\rm{O}}}}}}$$

2 $${{{{{{\rm{CH}}}}}}}_{3}{{{{{\rm{OH}}}}}}+{{{{{\rm{Olefin}}}}}}\to {{{{{\rm{HCHO}}}}}}+{{{{{\rm{Alkane}}}}}}$$

3 $${{{{{{\rm{CH}}}}}}}_{3}{{{{{\rm{OH}}}}}}\to {{{{{\rm{HCHO}}}}}}+{{{{{{\rm{H}}}}}}}_{2}$$

**Fig. 2:**
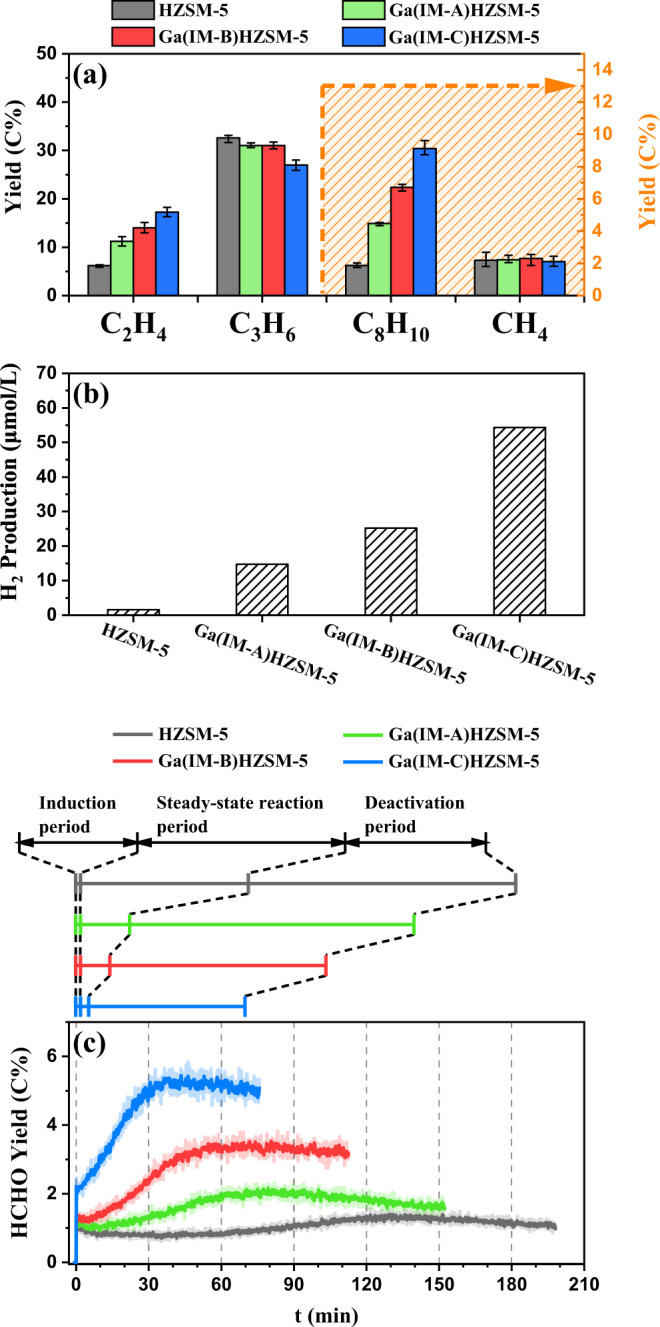
Product comparation over various Ga contents. **a** Yields of C_2_H_4_, C_3_H_6_, C_8_H_10_, CH_4_, (**b**) H_2_ production and (**c**) the real-time yields (C%) of HCHO in MTH reaction over parent HZSM-5 and various Ga(IM)HZSM-5. Reaction conditions of SR-PIMS experiments: 400 °C; MeOH WHSV = 12.52 g_MeOH_g_catalyst_^−1^h^−1^; and P = 2 Torr. C_3_H_6_ = propylene; and C_8_H_10_ = C_8_ aromatics. The yields in (**a**) were obtained during the steady-state reaction period. The H_2_ production of the catalyst samples in (**b**) was obtained under atmospheric pressure, which was carried out by GC‒MS. Each reaction in (**c**) proceeded until the C_2_H_4_ yield dropped to 0.5 C%. In (**c**), the original yield curves (transparent solid lines) have been smoothed as bright solid lines. Error bars obtained from repeated three sets of experiments on the same catalyst.

According to the yields of main MTH products over the parent HZSM-5 and Ga(IM-A/B/C)HZSM-5 (Fig. 2a and Supplementary Table 1), Ga(IM)HZSM-5 did not produce more methane than the parent HZSM-5. In addition, the HCHO yield remained high even when the catalysts were almost completely deactivated and nearly no olefins were generated (Fig. 1), under which hydrogen transfer from MeOH to olefins was negligible. It suggested that the increment of HCHO did not originate from the pathway of MeOH disproportionation and hydrogen transfer. Hence, the more reasonable pathway to generate the increased HCHO yield over Ga(IM)HZSM-5 is the direct dehydrogenation of MeOH (Eq. (3)), which can be supported by the concomitant increase in hydrogen production. The production of both H_2_ and HCHO was promoted with increasing Ga loading (Fig. 2b, c). The strong correlations between HCHO, H_2_, C_2_H_4_ and aromatics (Supplementary Fig. 6) depicted a clear reaction network, i.e., the direct dehydrogenation of MeOH over Ga(IM)HZSM-5 enhanced the HCHO-induced aromatic cycle. A similar mechanism was also observed over Zn/H-ZSM-5 prepared by Zn impregnation^29^.

### Determination of active sites

To determine the exact active sites that catalyze the dehydrogenation of MeOH, a series of characterizations were conducted. Through the impregnation method, Ga atoms can hardly be incorporated into the zeolite framework, and the main Ga species on Ga(IM)HZSM-5 are considered to be Ga_2_O_3_ and cationic Ga species (GaO^+^ or hydrated GaO^+^ ions)^7,30^. The FT-IR spectra of Ga-modified HZSM-5 presented a new peak at 3666 cm^−1^, which could be ascribed to GaOH groups^31^, implying the existence of extra-framework Ga species, such as GaO(OH) and highly dispersed Ga_2_O_3_ (Supplementary Fig. 8). Nanoscale Ga_2_O_3_ nanoparticles with uniform size distributions were also observed over HZSM-5 matrix, as shown in TEM images (Supplementary Fig. 9). In contrast, the lack of GaOH peak in FT-IR spectra and Ga_2_O_3_ particles in TEM for Ga(IE)HZSM-5 demonstrated that cationic GaO^+^ was predominant after washing with plenty of deionized water. As demonstrated in Table 1, both NH_3_-TPD and FT-IR of pyridine adsorption were conducted to reveal the acidic properties of the parent HZSM-5 and Ga-modified HZSM-5 zeolites. The NH_3_-TPD patterns presented three desorption peaks, located at 125 °C, 223 °C and 450 °C, which could be assigned to weak, medium and strong acid sites, respectively (Supplementary Fig. 10). The total acid amount initially decreased and then remained almost unchanged with increasing Ga loading. A similar trend of BAS indicated that ion exchange occurred between Ga species and HZSM-5. The ion exchange capacity might level off when the Ga content reached 2.4 wt%. More LAS were generated at the expense of BAS, suggesting the appearance of new acid sites that might be cationic GaO^+^ or Ga(OH)^2+^ species^12^. The systematic characterizations confirmed that Ga_2_O_3_ and cationic GaO^+^ coexisted in Ga(IM)HZSM-5. The dominant Ga species should be Ga_2_O_3_, after all the saturated exchange capacity of GaO^+^ was only 0.2 wt%.

To study the catalytic performance of BAS, Ga_2_O_3_ and cationic GaO^+^, MeOH was directly passed through Ga(IM-B)Silicalite-1 (with 2.5 wt.% Ga loading), single Ga_2_O_3_ and Ga(IE)HZSM-5, respectively. The formation of HCHO markedly increased, but none of hydrocarbon products were detected over Ga(IM-B)Silicalite-1, indicating that Brønsted acid sites are indispensable to the MTH process (Supplementary Fig. 12). A considerable amount of HCHO was produced, and dimethyl ether was also generated over pure Ga_2_O_3_ (Supplementary Fig. 13). Moreover, hydrogen was detected by GC‒MS experiments under atmospheric pressure (Supplementary Table 2). The above results indicated that Ga_2_O_3_ can dehydrogenate MeOH to HCHO with the release of H_2_^32^. In MeOH conversion over Ga_2_O_3_/HZSM-5 (prepared by physically mixing Ga_2_O_3_ and HZSM-5), although Hutchings et al. suggested that the active sites for promoting aromatic production were located in the Ga_2_O_3_/zeolite interface region^11^, the comparative experiments over Ga_2_O_3_/HZSM-5 with and without Y_2_O_3_ indicated that the increase in aromatic selectivity in MTH over Ga_2_O_3_/HZSM-5 is mainly due to the more HCHO generated from the dehydrogenation of MeOH over Ga_2_O_3_ (Supplementary Fig. 14 and Supplementary Table 2). However, Ga(IE)HZSM-5 exhibited the opposite behavior compared with Ga(IM)HZSM-5 under low-pressure conditions. Ga(IE)HZSM-5 produced even less HCHO than HZSM-5, leading to lower yields of aromatics. However, Ga(IM-D)HZSM-5 with 0.2 wt.% Ga loading still showed a slight promoting effect on HCHO and aromatic formation (Supplementary Fig. 15). This result firmly excluded the role of cationic GaO^+^ in dehydrogenating MeOH to HCHO. The lower HCHO over Ga(IE)HZSM-5 could be attributed to the decreased acid sites for disproportionation of MeOH or hydrogen transfer, resulting in impairing aromatic cycle^33^. In other words, this result strongly indicates that Ga_2_O_3_ should be assigned as the active phase in Ga(IM)HZSM-5 for the dehydrogenation of MeOH to HCHO. It should be noted that Ga(IE)HZSM-5 produced more ethene and aromatics, as well as H_2_, than HZSM-5 under atmospheric pressure experiments (Supplementary Table 2). There should exist another pathway for aromatic formation that involves the role of cationic GaO^+^. Thus, the LAS-induced aromatic formation pathway could be confirmed. Lewis acid sites (GaO^+^ species) cooperated with Brønsted acid sites to promote the dehydrogenation of alkenes and further contributed to the formation of aromatics^12,34,35^. The fast desorption of alkenes from catalysts under low pressure may explain the inconsistent aromatic selectivity for Ga(IE)HZSM-5 reacting at 2 Torr and atmospheric pressure. The LAS-induced dehydrogenation pathway was diminished to a negligible level during the SR-PIMS experiments.

### Transformation mechanism after reduction‒oxidation treatment

**Fig. 3:**
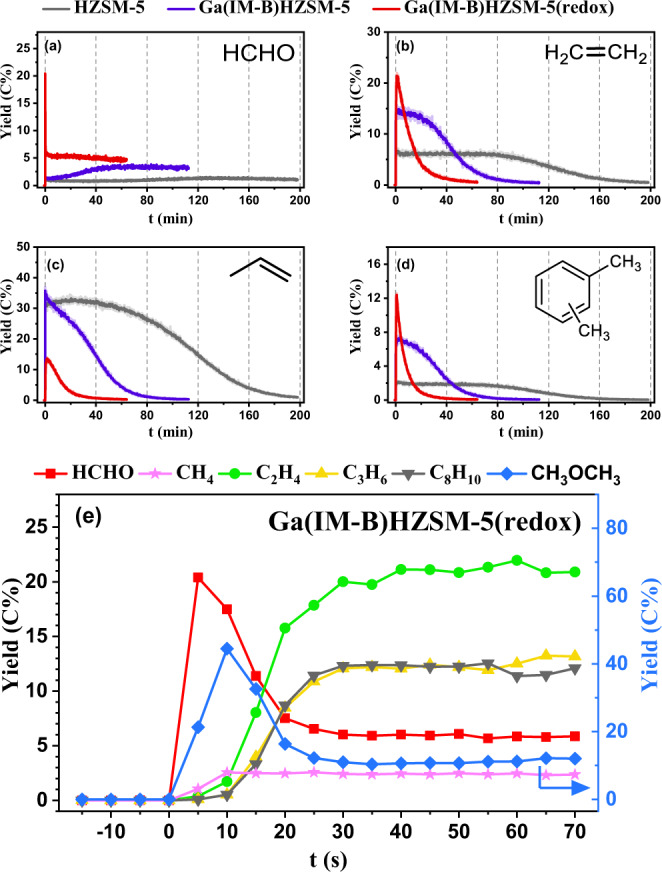
Product trend after reduction-oxidation treatment of Ga(IM)HZSM-5. The real-time yields (C%) of (**a**) HCHO, (**b**) C_2_H_4_, (**c**) propylene, and (**d**) C_8_ aromatics in MTH reaction over parent HZSM-5, Ga(IM-B)HZSM-5 and Ga(IM-B)HZSM-5(redox). **e** Is an enlarged view of the first 70 s in MTH over Ga(IM-B)HZSM-5(redox). Reaction conditions: 400 °C; MeOH WHSV = 12.52 g_MeOH_g_catalyst_^−1^h^−1^; P = 2 Torr; each reaction proceeded until the C_2_H_4_ yield dropped to 0.5 C%. The solid line in (**a**) is the original yield curve. In (**b**–**d**), the original yield curves (transparent solid lines) have been smoothed as bright solid lines. The product yields in the gray dotted box of (**e**) were the first real-time yields of products obtained after methanol feeding.

In previous works, HZSM-5 modified by a wetness impregnation method was further subjected to reduction‒oxidation treatment, and the sample showed higher aromatic selectivity and released more H_2_^7^. In this work, Ga(IM-B)HZSM-5 was further treated under H_2_-O_2_ flow, and the resultant sample was denoted as Ga(IM-B)HZSM-5(redox). Compared with Ga(IM-B)HZSM-5, Ga(IM-B)HZSM-5(redox) did produce more aromatics in the first few minutes of the MTH reaction (Fig. 3 and Supplementary Fig. 16). An interesting phenomenon was observed in which the HCHO yield changed significantly in MTH over Ga(IM-B)HZSM-5(redox) (Fig. 3a). Different from the time-resolved profiles of HCHO over Ga(IM)HZSM-5 without reduction‒oxidation treatment, the HCHO yield of Ga(IM-B)HZSM-5(redox) reached the maximum value at the moment after feeding MeOH, and then rapidly decreased to a stable yield within 30 seconds due to the competitive catalytic reactions through the rapidly formed hydrocarbon pool (HCP), as shown in Fig. 3e. After that, the HCHO yield remained stable at a high value, and only slightly decreased before deactivation.

**Fig. 4:**
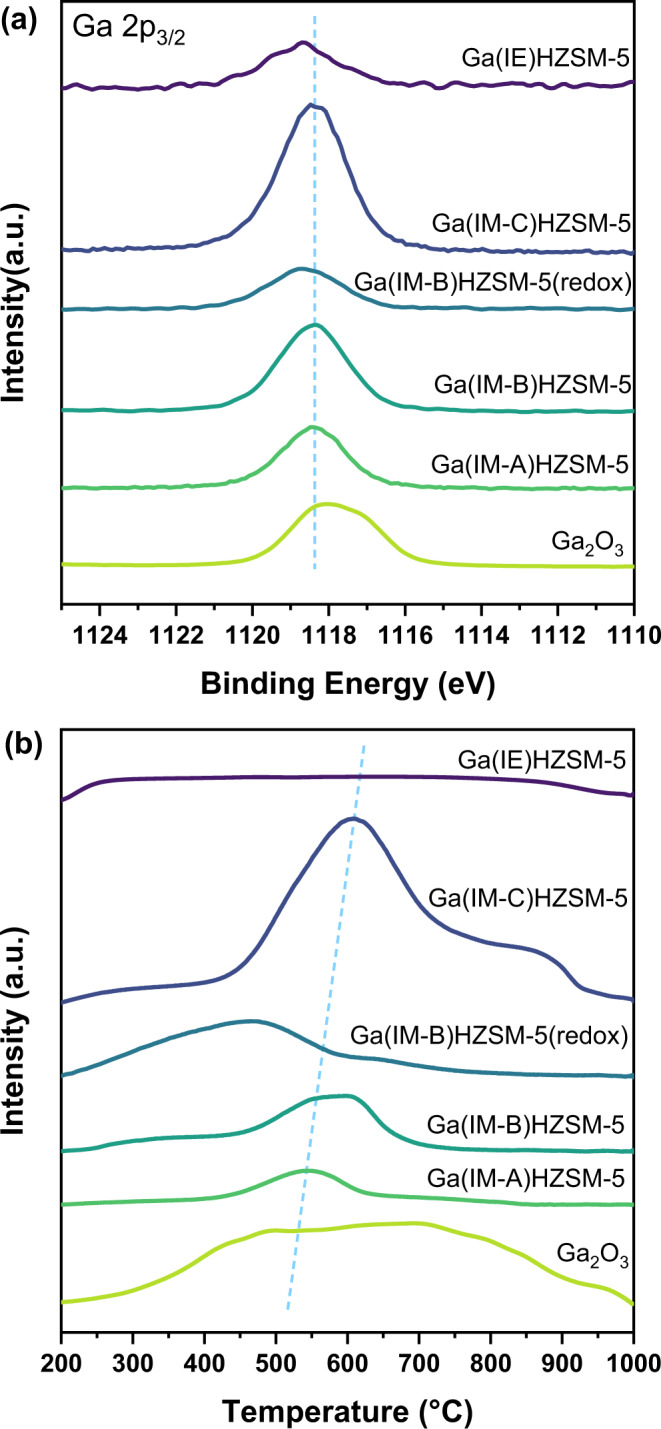
Surface species and reductive properties of Ga-containing samples. **a** XPS Ga 2p_3/2_ spectra of pure Ga_2_O_3_ and Ga-modified HZSM-5. **b** H_2_-TPR profiles of pure Ga_2_O_3_ and Ga-modified HZSM-5.

**Fig. 5:**
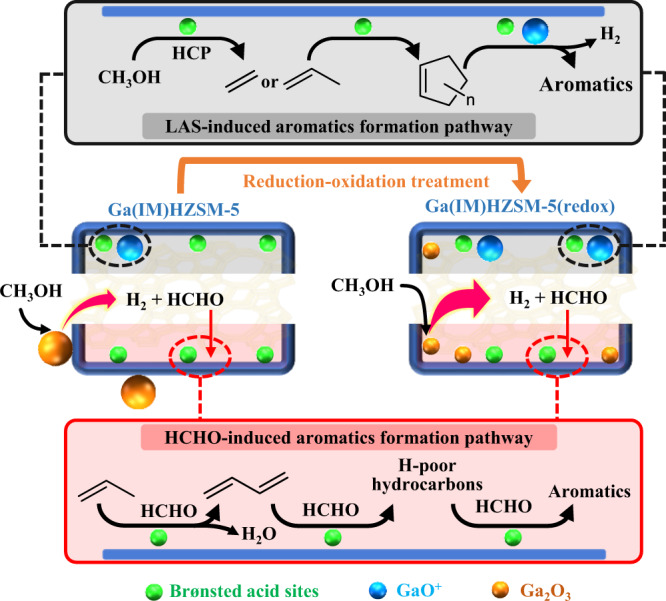
Proposed evolution mechanisms of catalyst and aromatic formation. Mechanistic pathways for the formation of HCHO on Ga_2_O_3_, migration and transformation of Ga species upon reduction‒oxidation treatment, and the two kinds of aromatic formation pathways.

The acid properties of Ga(IM-B)HZSM-5(redox) presented significant decrease in strong acid sites and BAS, along with distinct increase in weak and medium acid sites and LAS (Table 1)^36^. This result was consistent with the reported phenomenon that Ga_2_O_3_ particles located on the external surface of zeolite crystallites could be transformed into highly dispersed cationic Ga species after reduction‒oxidation treatment^37,38^. The XPS spectra of Ga(IM-B)HZSM-5(redox) displayed much lower surface Ga intensity and higher binding energy, similar to Ga(IE)HZSM-5, further proving the migration of gallium from the external surface of the zeolite crystallites to their intracrystalline volume (Fig. 4a)^39^. The H_2_-TPR profiles showed huge differences in reductive properties for various Ga-based samples (Fig. 4b). There was a broad peak for pure Ga_2_O_3_. The main peak near 600 °C for Ga(IM)HZSM-5 could be assigned to the reduction of well-dispersed Ga_2_O_3_ particles, which shifted to higher temperature with higher Ga loading, consistent with the larger Ga_2_O_3_ particles over Ga(IM-C)HZSM-5 observed from TEM images. The peaks over 800 °C could be attributed to the reduction of large and bulk Ga_2_O_3_ particles separated from the zeolite matrix. No reduction peak was observed from Ga(IE)HZSM-5 in the profiles, which revealed that Ga species sitting at the cationic locations could hardly be reduced by H_2_^40^. Remarkably, the reduction peak of Ga(IM-B)HZSM-5(redox) shifted toward much lower temperature, indicating the generation of smaller Ga_2_O_3_ particles through redox treatment. Combined with the fact that cationic GaO^+^ had no activity to generate HCHO, we could attribute the sharp increase in HCHO over Ga(IM-B)HZSM-5(redox) to the redispersed Ga_2_O_3_ active phase entering the zeolite channels. In addition, another route to aromatic formation that originated from the dehydrogenation of alkenes would also be enhanced due to the much higher LAS concentration for Ga(IM-B)HZSM-5(redox) (Supplementary Table 2). It demonstrated that both the LAS-induced and HCHO-induced aromatic formation pathways coexisted over Ga(IM)HZSM-5. The former pathway involved cationic GaO^+^ species, and the latter pathway took Ga_2_O_3_ as the active phase (Fig. 5). GaO^+^ species combined with adjacent BAS promoted the dehydrogenation process of cycloalkenes and cycloalkanes to higher unsaturated hydrocarbons, up to aromatics. Formaldehyde reacted with alkenes at BAS to form dienes and then react stepwise to form H-poor hydrocarbons and eventually aromatics (Supplementary Fig. 17).

## Conclusions

In this work, we studied the MTH reaction over Ga-modified HZSM-5 with the advantage of in situ SR-PIMS. Some new insights are provided here. 1) The HCHO-induced aromatic formation pathway promoted by Ga(IM)HZSM-5 producing more HCHO was found to play an important role in increasing aromatic yields. 2) More HCHO was attributed to the direct dehydrogenation of MeOH, and Ga_2_O_3_ in Ga-modified HZSM-5 was confirmed to be the active center. 3) Cationic GaO^+^ enhanced alkenes dehydrogenation and Ga_2_O_3_ promoted methanol dehydrogenation process, which contributed together to aromatic formation. 4) Upon reduction‒oxidation treatment, redispersed Ga_2_O_3_ with smaller sizes in Ga(IM)HZSM-5(redox) was found to further enhance MeOH dehydrogenation reaction, and therefore produce more HCHO at a faster rate. Simultaneously, the significant increase in LAS concentration after redox treatment promoted alkene dehydrogenation-aromatization processes. The above perspectives supplement the single LAS-induced aromatic formation mechanism with new insights into the HCHO-induced aromatic formation pathway over Ga-modified zeolites and provide a new thought to manipulate the MTH reaction.

## Methods

### Catalyst preparations

HZSM-5 (SiO_2_/Al_2_O_3_ = 36) was purchased from Nankai University Catalyst Co., Ltd. (Tianjing, China). Ga(IM)HZSM-5 was prepared by impregnation method. In the preparation, 6.0 g of HZSM-5 was added into 10 mL of Ga(NO_3_)_3_ solutions containing 0.2, 0.5, 1.0 and 0.04 g of Ga(NO_3_)_3_·xH_2_O. The resulting mixed solution was continuously stirred at 50 °C until it became dry. The dry mixture was subsequently dried in flowing air (50 sccm) at 110 °C for 6 h, and then calcined in flowing air (50 sccm) at 550 °C for 5 h. The three Ga(IM)HZSM-5 samples with different Ga loadings were denoted as Ga(IM-A)HZSM-5, Ga(IM-B)HZSM-5, Ga(IM-C)HZSM-5 and Ga(IM-D)HZSM-5, respectively. The same procedure as Ga(IM-B)HZSM-5 was applied for preparation of Ga(IM-B)Silicalite-1. The prepared Ga(IM)HZSM-5 was further treated under 50 sccm of pure hydrogen at 450 °C for 1 h at a heating rate of 10 °C min^-1^, and then under 50 sccm of dry air at 450 °C for 1 h. The obtained sample was denoted as Ga(IM)HZSM-5(redox). Ga(IE)HZSM-5 was prepared by ion exchange method. In total, 3.0 g of HZSM-5 was dispersed in 50 mL of 1 M Ga(NO_3_)_3_ solution. After stirring at 80 °C for 4 h, the zeolite was filtered and washed with large amount of deionized water, followed by drying at 100 °C for 6 h. The above steps were repeated three times. Finally, the resulting sample was calcined at 550 °C for 5 h at a heating rate of 1 °C min^-1^. Ga(IM)HZSM-5/Y_2_O_3_ was prepared by physically mixing the prepared Ga(IM)HZSM-5 with Y_2_O_3_ at a mass ratio of 1:1 using an agate mortar and pestle. Ga_2_O_3_/HZSM-5 and Ga_2_O_3_/HZSM-5/Y_2_O_3_ were also prepared by physical mixing method. The mass ratio of Ga_2_O_3_ and HZSM-5 in Ga_2_O_3_/HZSM-5 is 0.2:3, and the mass ratio of Ga_2_O_3_, HZSM-5 and Y_2_O_3_ in Ga_2_O_3_/HZSM-5/Y_2_O_3_ is 0.2:3:3. All kinds of powder samples were pelletized, crushed and sieved to 30-40 mesh before catalytic testing.

### Catalyst characterizations

The Ga contents in the prepared samples were determined by an inductively coupled plasma-atomic emission spectroscopy (ICP‒AES) method using Thermo iCAP 7600 equipment. In a typical test, 50 mg of zeolite sample was dissolved in the mixture of 2 mL of HClO_4_, 8 mL of HF and 4 mL of HNO_3_, and then diluted to 250 mL with deionized water. Powder X-ray diffraction (XRD) patterns were obtained on a Smartlab diffractometer using Cu Kα radiation at 40 KV and 150 mA. Transmission electron microscopy (TEM) images were obtained on a JEOL JEM-2011 instrument operating at 200 KV. Nitrogen adsorption/desorption isotherms were tested at −196 °C on a TriStar II 3020 M system. Before measurement, the samples were degassed at 300 °C for 30 min under vacuum conditions. The specific surface area was determined by the Brunauer‒Emmett‒Teller (BET) method, and the pore volume was calculated by the t-Plot method. X-ray photoelectron spectra (XPS) were recorded with an ESCALAB 250Xi spectrometer. The FT-IR experiments of the catalysts, pressed into tablets with KBr before scanning, were performed by Nicolet 8700. The zeolite/KBr tablet was heated at 105 °C for 12 h to remove water prior to analysis. Temperature-programmed reduction (H_2_-TPR) was measured by a VDSorb-91i-VAP-HB chemical adsorption instrument. Before testing, 100 mg of catalyst was heated in Ar flow at 500 °C for 1 h at a heating rate of 10 °C min^-1^. Afterward, the TPR test was performed from 200 °C to 1000 °C at a heating rate of 10 °C min^-1^ in a mixture of 10% H_2_/Ar flow. NH_3_ temperature-programmed desorption (NH_3_-TPD) was also employed on a VDSorb-91i-VAP-HB chemical adsorption instrument. Typically, 100 mg of sample was pretreated at 500 °C for 1 h at a heating rate of 10 °C min^-1^ under He flow. After cooling to 40 °C, 1% NH_3_/He flow was introduced and adsorbed for 1 h, followed by purging in He flow for another 1 h to remove the physisorbed NH_3_. Subsequently, a temperature program was performed from 40 °C to 800 °C at a heating rate of 10 °C min^-1^. The FT-IR spectra of pyridine adsorption were recorded by a Nicolet 5700 FTIR spectrometer. In total, 15 mg of catalyst sample was pressed into self-supporting wafers (R. 6.5 mm) and pretreated in vacuum at 400 °C for 2 h at a heating rate of 10 °C min^-1^. Then, they were exposed to excessive amount of pyridine and further evacuated at 200 °C for 1 h. After that, the system was heated to 200 °C, 300 °C and 400 °C under vacuum, and the IR spectra were recorded. The concentrations of Brønsted acid sites (BASs) and Lewis acid sites (LASs) were quantified from the IR spectra at 200 °C and based on the band areas at 1510–1564 cm^−1^ and 1421–1466 cm^−1^, respectively.

### Catalytic testing

Catalytic reactions were carried out under both low-pressure and atmospheric pressure conditions. To capture the intermediately formed formaldehyde, an in situ low-pressure catalytic reactor combined with in situ synchrotron radiation photoionization mass spectrometry (SR-PIMS) was employed to perform the catalytic conversion of methanol. The co-feeding of formaldehyde was conducted by the mixture of 5 wt.% trioxane in methanol^41^. The low-pressure catalytic experiment apparatus mainly included a bubbler feeding system, a low-pressure catalytic reactor and a homemade orthogonal time-of-flight mass spectrometer (*oa* TOF-MS) (Supplementary Fig. 1). The ionization source used in the mass spectrometer was the synchrotron VUV light. The catalyst was placed in a quartz reactor (O.D. 8 mm, I.D. 6 mm, L. 150 mm) of the low-pressure catalytic reactor. A K-type thermocouple wrapped in a quartz tube was inserted adjacent to the catalyst. In this way, the temperature of the catalyst can be detected in real time. During the low-pressure experiment, the pressure of the catalytic reactor was maintained at 2 Torr, which was achieved by a closed-loop control system consisting of a pressure sensor, a butterfly valve (Model 61232-KEGG-0002, VAT, Switzerland) and a vacuum pump. For the atmospheric pressure experiment, the catalysts were placed in front of the quartz sand plate in a quartz reactor (O.D. 10 mm, I.D. 7 mm, L. 340 mm) (Supplementary Fig. 2), and quartz wool was placed before and after the catalysts for fixation. The effluent products flowed through the heated transfer line into GC‒MS (Agilent 5977B GC/MSD & 8890 GC System) for online analysis. The transfer line was heated to 200 °C to avoid condensation of volatile products. In this GC‒MS, the thermal conductivity detector (TCD) equipped with a HayeSep Q column was used for inorganic product analysis, and the flame ionization detector (FID) equipped with a HP-PLOT/Q capillary column was used for organic compound analysis. In the catalytic testing, 150 mg of samples for HZSM-5, Ga(IM)HZSM-5, single Ga_2_O_3_, Ga(IM)HZSM-5(redox), Silicalite-1 and Ga(IM)Silicalite-1 were used. To ensure that the amounts of the zeolite contained in the physically mixed samples were the same, 300 mg of Ga(IM)HZSM-5/Y_2_O_3_, 160 mg of Ga_2_O_3_/HZSM-5 and 310 mg of Ga_2_O_3_/HZSM-5/Y_2_O_3_ were used, respectively. Before the catalytic reaction, the catalysts were pretreated in Ar flow (250 sccm) at 500 °C for two hours at a heating rate of 10 °C min^-1^.

## Supplementary information

Pan_PR File

Supplementary information

## Data Availability

All results are reported in the main paper and Supplementary information. All other data are available from the authors upon request.
