# Supplementary material for: Role of formaldehyde in promoting aromatic selectivity during methanol conversion over gallium-modified zeolites
Source: Commun Chem. 2022 Nov 19;5:153. doi: 10.1038/s42004-022-00771-8 (PMC9814038; doi:10.1038/s42004-022-00771-8)
Supplement: Supplementary file 2 — Supplementary information [file 42004_2022_771_MOESM2_ESM.pdf]

Role of formaldehyde in promoting aromatic selectivity during methanol conversion over gallium-modified zeolites

Wu Wen[1,5], Tianci Xiao[1,5], Beibei Feng[1], Chaoqun Zhou[2], Jian Li[3], Hao Ma[2], Zhongyue Zhou[2], Ying Zhang[4], Jiuzhong Yang[1], Zhandong Wang[1], Fei Qi[2], Jun Bao[1], Chengyuan Liu[1]* and Yang Pan[1]*

[1] National Synchrotron Radiation Laboratory, University of Science and Technology of China, Hefei 230029, Anhui (P. R. China).

[2] Key Laboratory for Power Machinery and Engineering of Ministry of Education, Shanghai Jiao Tong University, Shanghai 200240 (P. R. China).

[3] Shanghai Research Institute of Petrochemical Technology SINOPEC, Shanghai 201208 (P. R. China).

[4] Department of Chemistry, University of Science and Technology of China, Hefei 230029, Anhui (P. R. China).

[5] These authors contributed equally: Wu Wen, Tianci Xiao.

* e-mail: lcy2012@ustc.edu.cn; panyang@ustc.edu.cn

Table of Contents

1. **Supplementary Methods**

   1.1 Materials
   1.2 In situ synchrotron radiation photoionization mass spectrometry experiment under low pressure
   1.3 GC-MS experiment under atmospheric pressure

2. **Supplementary Note 1_Calculation formulas of methanol conversion and product yield**
3. **Supplementary Note 2_Division of the three periods in MTH reaction**
4. **Supplementary figure in MTH over HZSM-5, Ga(IM-A)HZSM-5 and Ga(IM-A)HZSM-5/Y_2O_3**
5. **Supplementary graph in MTH with or without co-feeding HCHO over HZSM-5**
6. **Supplementary graph in relationship between H_2/HCHO/hydrocarbon production and Ga content**
7. **Supplementary figure of catalyst characterizations**
8. **Supplementary mass spectrum in MTH over Silicalite-1 and Ga-modified Silicalite-1**
9. **Supplementary mass spectrum in MTH over single Ga_2O_3**
10. **Supplementary figure in MTH over HZSM-5, Ga_2O_3/HZSM-5 and Ga_2O_3/HZSM-5/Y_2O_3**
11. **Supplementary figure in MTH over HZSM-5 and Ga(IE)HZSM-5**
12. **Supplementary figure in MTH over HZSM-5, Ga(IM-B)HZSM-5 and Ga(IM-B)HZSM-5(redox)**
13. **Supplementary detailed reaction routes of aromatics formation**
14. **Detailed results of MTH reaction under low pressure obtained by SR-PIMS experiments**
15. **Detailed results of MTH reaction under atmospheric pressure obtained by GC-MS**
16. **Supplementary references**

1. Supplementary Methods

1.1 Materials

HZSM-5 (SiO_2/Al_2O_3 = 36) and Silicalite-1 were purchased from Nankai University Catalyst Co., Ltd (Tianjing, China). $Ga(NO_3)_3 \cdot xH_2O$ (99.99% metals basis) was purchased from Shanghai Macklin Biochemical Co., Ltd. Ga_2O_3 (99.999% metals basis) was purchased from Shanghai Aladdin Biochemical Technology Co., Ltd. Y_2O_3 (purity ≥ 99.99%) was purchased from Sinopharm Chemical Reagent Co., Ltd. Methanol (purity ≥ 99.98%) was purchased from TEDIA Co., Ltd. 1,3,5-trioxane (purity ≥ 99.5%) was purchased from Shanghai Macklin Biochemical Co., Ltd. Ar (purity ≥ 99.999%), N_2 (purity ≥ 99.999%), He (purity ≥ 99.999%) and high purity synthetic air were purchased from Hefei Ningte Gas Management Co., Ltd. Hydrogen was generated by the hydrogen generator (Peak Scientific Instruments Ltd.).

1.2 In situ synchrotron radiation photoionization mass spectrometry experiment under low pressure

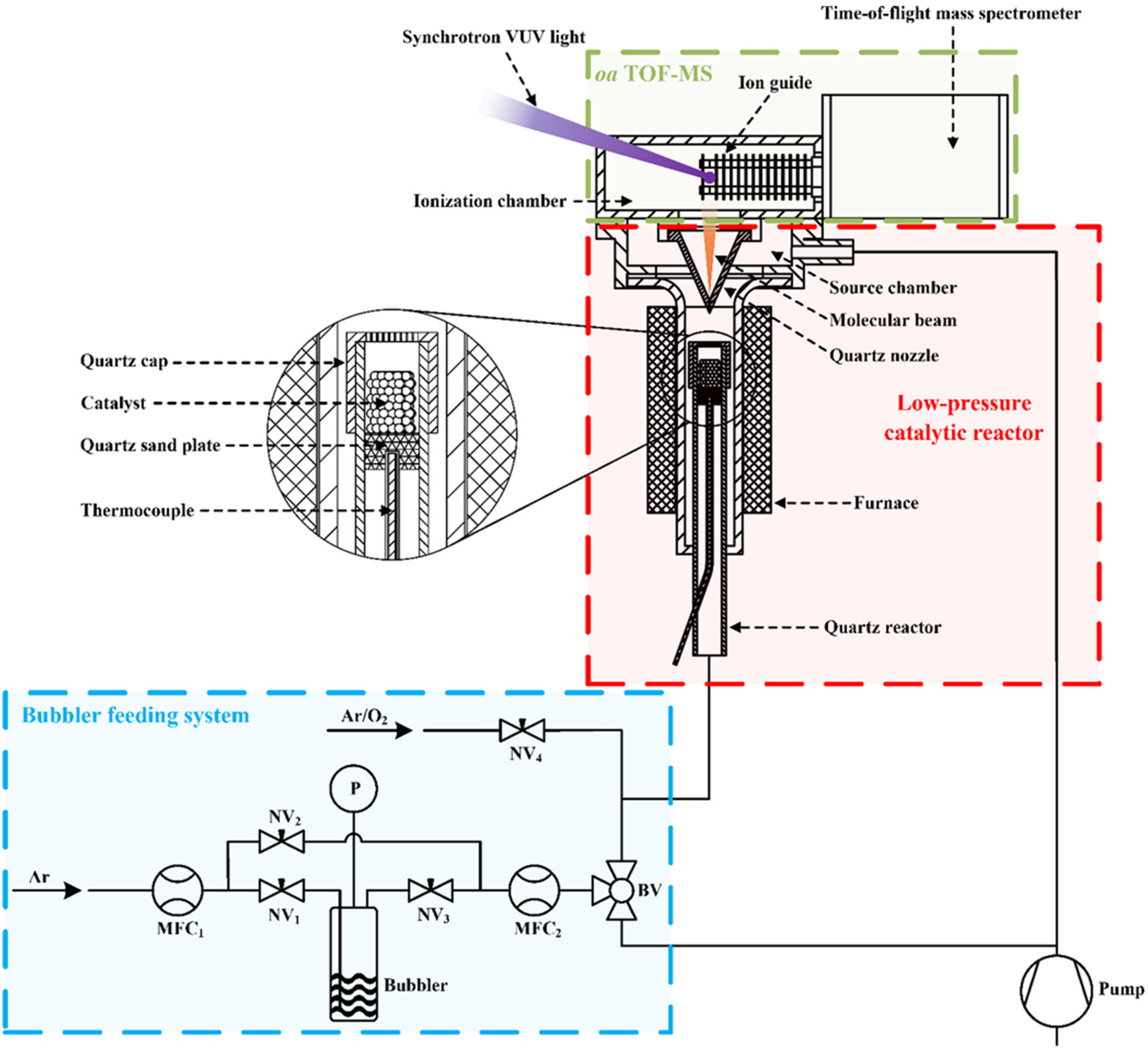

Supplementary Fig. 1. The low-pressure catalytic experiment apparatus consisted of a bubbler feeding system, a low-pressure catalytic reactor and a homemade orthogonal time-of-flight mass spectrometer (oa TOF-MS) with a synchrotron vacuum ultraviolet (VUV) light as ionization source.

In order to capture the intermediately formed formaldehyde, an in situ low-pressure catalytic reactor combined with in situ synchrotron radiation photoionization mass spectrometry (SR-PIMS) was employed to perform the catalytic conversion of methanol. The SR-PIMS experiments were carried out at the mass spectrometry end-station of the National Synchrotron Radiation Laboratory (NSRL) in Hefei, China. The details of the beamline and the low-pressure catalytic experiment apparatus have been reported elsewhere, so only a brief description is given here.[1,2]

The synchrotron vacuum ultraviolet (VUV) light was from an undulator-based beamline (BL03U), in which the monochromator was equipped with a laminar grating (Horiba Jobin Yvon, France) with line density of 200 lines mm^{-1}. The grating covered the photon energy from 7.5 to 22 eV, and its energy resolving power was 3000 ($E/\Delta E$ @ 10 eV). The higher order harmonic radiation was suppressed by a gas filter filled with argon. The average photon flux of this beamline can reach a magnitude of 10^{13} photons/s. The photon flux, which was used to normalize the ion signals, was monitored by a silicon photodiode (SXUV-100, International Radiation Detectors, Inc., U.S.A).

The low-pressure catalytic experiment apparatus mainly included a bubbler feeding system, a low-pressure catalytic reactor and a homemade orthogonal time-of-flight mass spectrometer (*oa* TOF-MS) (Supplementary Fig. 1.). The ionization source used in the mass spectrometer was the synchrotron VUV light. Based on the tunable characteristic of synchrotron VUV light, the near-threshold photoionization of molecules without fragmentation can be achieved, and the isomers with different ionization energies can be distinguished by scanning their photoionization efficiency (PIE) spectra.[3,4] The catalyst was placed in the quartz reactor (O.D. 8 mm, I.D. 6 mm, L. 150 mm) of the low-pressure catalytic reactor. A K-type thermocouple wrapped in a quartz tube was inserted adjacent to catalyst. In this way, the temperature of the catalyst can be detected in real time. During the low-pressure experiment, the pressure of the catalytic reactor was maintained at 2 Torr, which was achieved by a closed-loop control system consisting of a pressure sensor, a butterfly valve (Model 61232-KEGG-0002, VAT, Switzerland) and a vacuum pump.

Prior to the catalytic testing, all kinds of catalyst samples were treated at 500 °C for 2 hours in a gas mixture of Ar (100 sccm) and O_2 (100 sccm). Then after the gas mixture was evacuated by the pump, the furnace temperature was cooled to the predetermined reaction temperature in pure Ar atmosphere (100 sccm), and the used Ar gas was introduced into reactor through the needle valve NV_4. In order to avoid the existence of oxygen in the bubbler, the residual gas in the bubbler was evacuated after the bubbler was frozen with liquid nitrogen. Then the methanol in the bubbler was thawed and returned to room temperature. In the bubbler feeding system, two mass flow controllers (MFCs) were connected on both sides of the bubbler. The 200 sccm of Ar, which was controlled by MFC_1, entered the methanol bubbler through the needle valve NV_1, and took out methanol vapor through the needle valve NV_3. The flow rate of the outflowing gas mixture controlled by MFC_2 was set to 250 sccm. The flow rate of methanol vapor was calculated according to the setting value of the two MFCs, not simply by the flow rate difference between the MFCs. The calculation method of methanol flow rate has been detailed in our previous study.[2] The methanol vapor flow rate obtained by calculation was 21.89 sccm. In order to ensure the stability of the methanol feeding before the catalytic reaction, the outflowing gas mixture directly entered the pump through the three-way ball valve BV, and that was kept for 30 minutes. When the catalytic reaction started, the outflowing gas mixture was introduced into the low-pressure catalytic reactor by switching the outlet of the three-way ball valve BV. A small fraction of the catalytic products would be sampled rapidly into the ionization chamber through a quartz nozzle (I.D. 450 μm). The molecular beam was ionized by the crossed synchrotron VUV light, and the generated ions were transmitted into the time-of-flight mass spectrometer by the ion guide. The ion signal was amplified with a VT120C preamplifier (EG&G, ORTEC, Oak Ridge, TN) and then recorded by a P7888-2 multiscaler (FAST Comtec, Oberhaching, Germany). In detecting the real-time yields of products, the acquisition time of a single spectrum was 5 seconds.

1.3 GC-MS experiment under atmospheric pressure

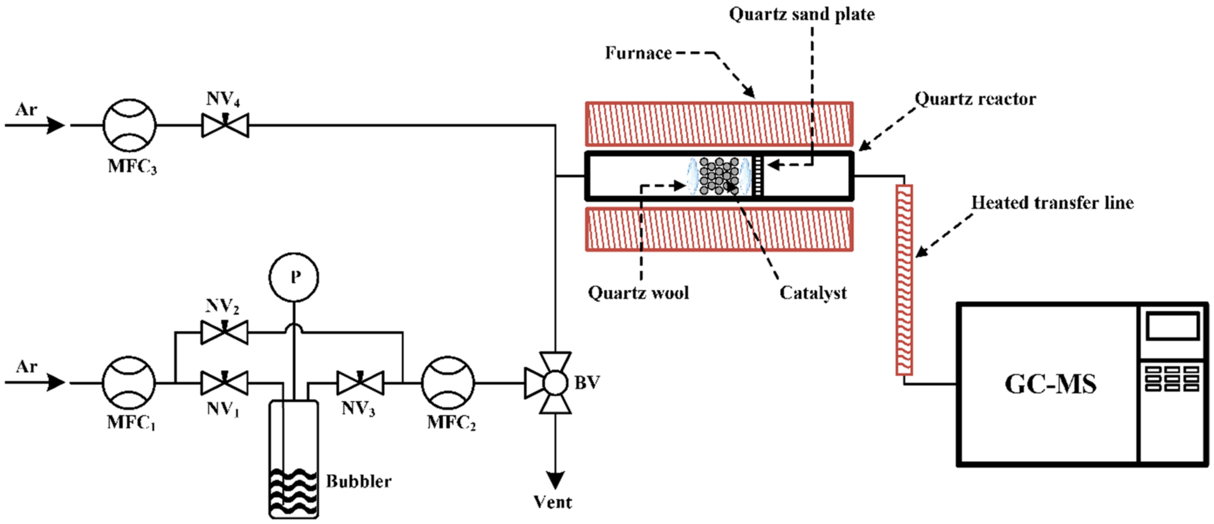

Supplementary Fig. 2. The catalytic experiment apparatus for MTH reaction under atmospheric pressure.

The catalysts were placed in front of the quartz sand plate in quartz reactor (O.D. 10 mm, I.D. 7 mm, L. 340 mm) (Supplementary Fig. 2.), and quartz wool was placed before and after the catalysts for fixation. Before the catalytic reaction, the needle valve NV_4 was opened, and the catalysts were pretreated in Ar flow (250 sccm) at 500 °C for two hours. The bubbler had been emptied of residual oxygen by the method of liquid nitrogen freezing described above. The 30 sccm of Ar, controlled by MFC_1, entered the methanol bubbler through the needle valve NV_1, and took out methanol vapor through NV_3. The flow rate of the outflowing gas mixture controlled by MFC_2 was set to 37 sccm. According to the calculation method in our previous study,[2] the flow rate of methanol was 3.07 sccm. In order to ensure the stability of methanol feeding before catalytic reaction, the outflowing gas mixture through the three-way ball valve BV was directly led to the vent and kept for 30 minutes. After the furnace was cooled to the predetermined reaction temperature, the Ar flow rate through MFC_3 was changed to 200 sccm, and the outlet of the three-way ball valve BV was switched to make the reactant gas enter the quartz reactor. The effluent products flowed through the heated transfer line into GC-MS (Agilent 5977B GC/MSD & 8890 GC System) for online analysis. The transfer line was heated to 200 °C to avoid condensation of volatile products. In this GC-MS, the thermal conductivity detector (TCD) equipped with a HayeSep Q column was used for inorganic product analysis, and the flame ionization detector (FID) equipped with a HP-PLOT/Q capillary column was used for organic compound analysis.

2. Supplementary Note 1_Calculation formulas of methanol conversion and product yield

In this work, methanol conversion, product selectivity, and product yield were calculated on a molar carbon basis. The methanol conversion can be calculated from the following formula:

$$X_{MeOH} = \frac{n_{MeOH_{in}} - n_{MeOH_{out}}}{n_{MeOH_{in}}} \cdot 100\% \tag{1}$$

X_{MeOH} is the methanol conversion; $n_{MeOH_{in}}$ is the molar amount of methanol flowing into the reactor; $n_{MeOH_{out}}$ is the molar amount of methanol flowing out of the reactor.

The selectivity of product is calculated based on the carbon number as follow:

$$S_i = \frac{N_{C,i} \cdot n_i}{n_{MeOH_{in}} - n_{MeOH_{out}}} \cdot 100\% \tag{2}$$

S_i is the selectivity of a product i; n_i is the molar amount of a product i generated from the MTH reaction; $N_{C,i}$ is the carbon number of a product i.

The yield (Y_i) of a product i can be calculated by its selectivity (S_i) and methanol conversion (X_{MeOH}):

$$Y_i = \frac{S_i \cdot X_{MeOH}}{100} \% \tag{3}$$

3. Supplementary Note 2_Division of the three periods in MTH reaction

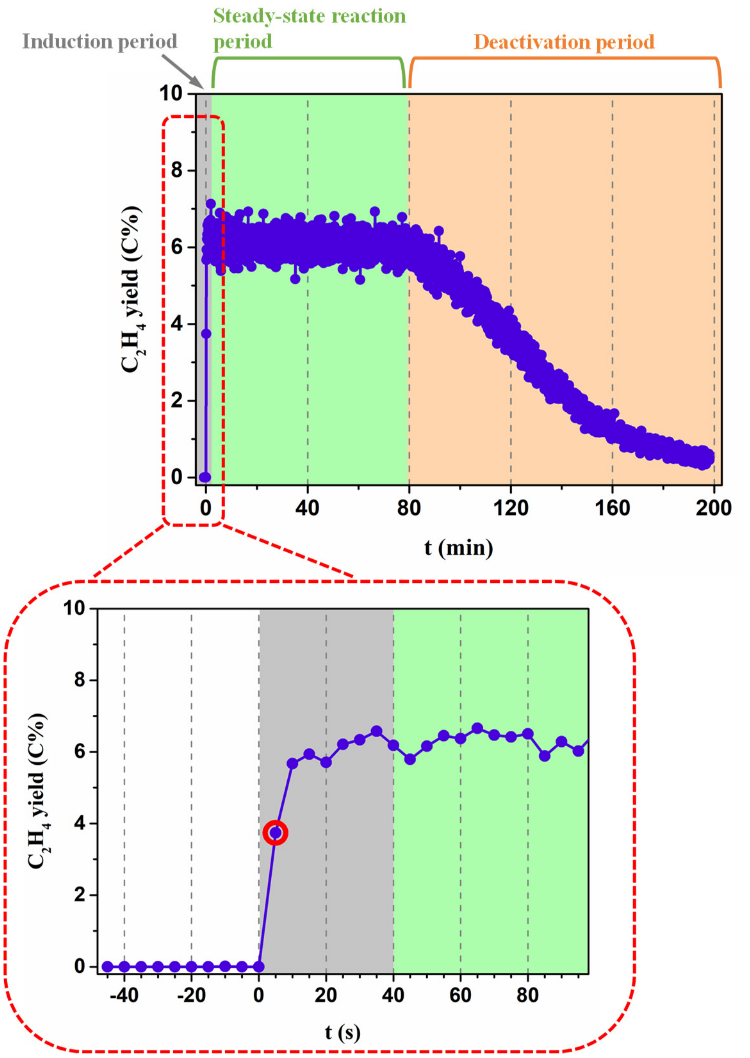

Supplementary Fig. 3. The division of the three periods in MTH reaction: taking the real-time yield of ethylene as an example. The magnified figure of the induction period is in the red dotted box. The red circle represents the first ethylene real-time yield collected after methanol feeding.

During the induction period just after methanol feeding, a series of initial hydrocarbons are generated in the zeolite pores through the first C-C bond formation mechanism.[5,6] The initial hydrocarbons will contribute to the formation of active hydrocarbon pool (HCP). The HCP, as a cocatalyst, and the acid sites of zeolites work together to catalyze the conversion of methanol into hydrocarbons, which is considered as an autocatalysis reaction process.[7] The increase in the amount and activity of HCP will further accelerate the formation of products. Therefore, the yields of hydrocarbons rise rapidly during the induction period, which is shown in the gray part of Supplementary Fig. 3. When the MTH enters the steady-state reaction period, since the HCP can hold steady for a period of time, the yields of hydrocarbons also remain relatively constant. As shown in the green part of Supplementary Fig. 3., the period during which the ethylene yield remains stable is the so-called steady-state reaction period.[8] With the formation and accumulation of cokes in zeolite pores, the catalyst conversion efficiency begins to decline,[9] which can be reflected by the gradual decrease in the yields of hydrocarbons. This indicates that the MTH has entered the deactivation period, which is shown in the orange part of Supplementary Fig. 3. In this work, when evaluating the performance of different catalyst samples, the conversion and product yield of each sample during the steady-state reaction period of MTH were selected.

4. Supplementary graph in MTH over HZSM-5, Ga(IM-A)HZSM-5 and Ga(IM-A)HZSM-5/Y_2O_3

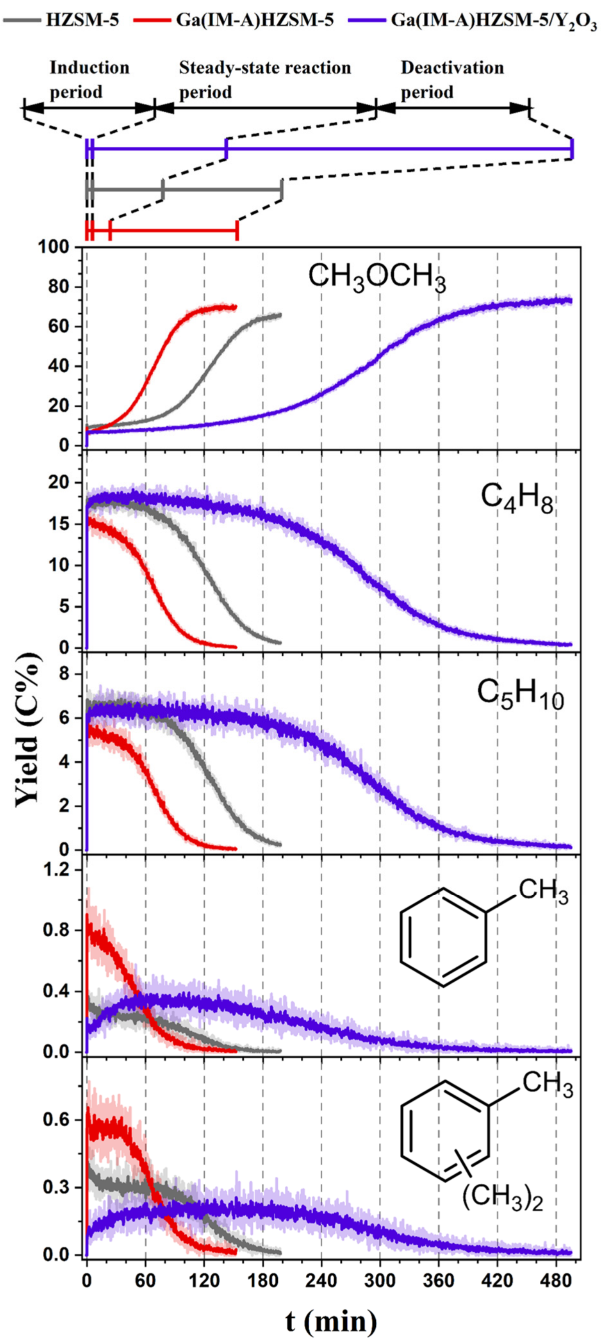

Supplementary Fig. 4. The real-time yields (C%) of CH_3OCH_3, C_4H_8, C_5H_{10}, C_7H_8 and C_9H_{12} in the MTH reaction over parent HZSM-5, Ga(IM-A)HZSM-5 and Ga(IM-A)HZSM-5/Y_2O_3. Reaction conditions: 400 °C; methanol WHSV = 12.52 $g_{MeOH}g_{catalyst}^{-1}h^{-1}$; P = 2 Torr; and each reaction proceeded until the ethylene yield dropped to 0.5 C%. The original yield curves (transparent solid lines) obtained in the experiments have been smoothed as bright solid lines. CH_3OCH_3 = dimethyl ether; C_4H_8 = $C_4^=$ olefins; C_5H_{10} = $C_5^=$ olefins; C_7H_8 = toluene; and C_9H_{12} = C_9 aromatics.

5. Supplementary graph in MTH with or without co-feeding HCHO over HZSM-5

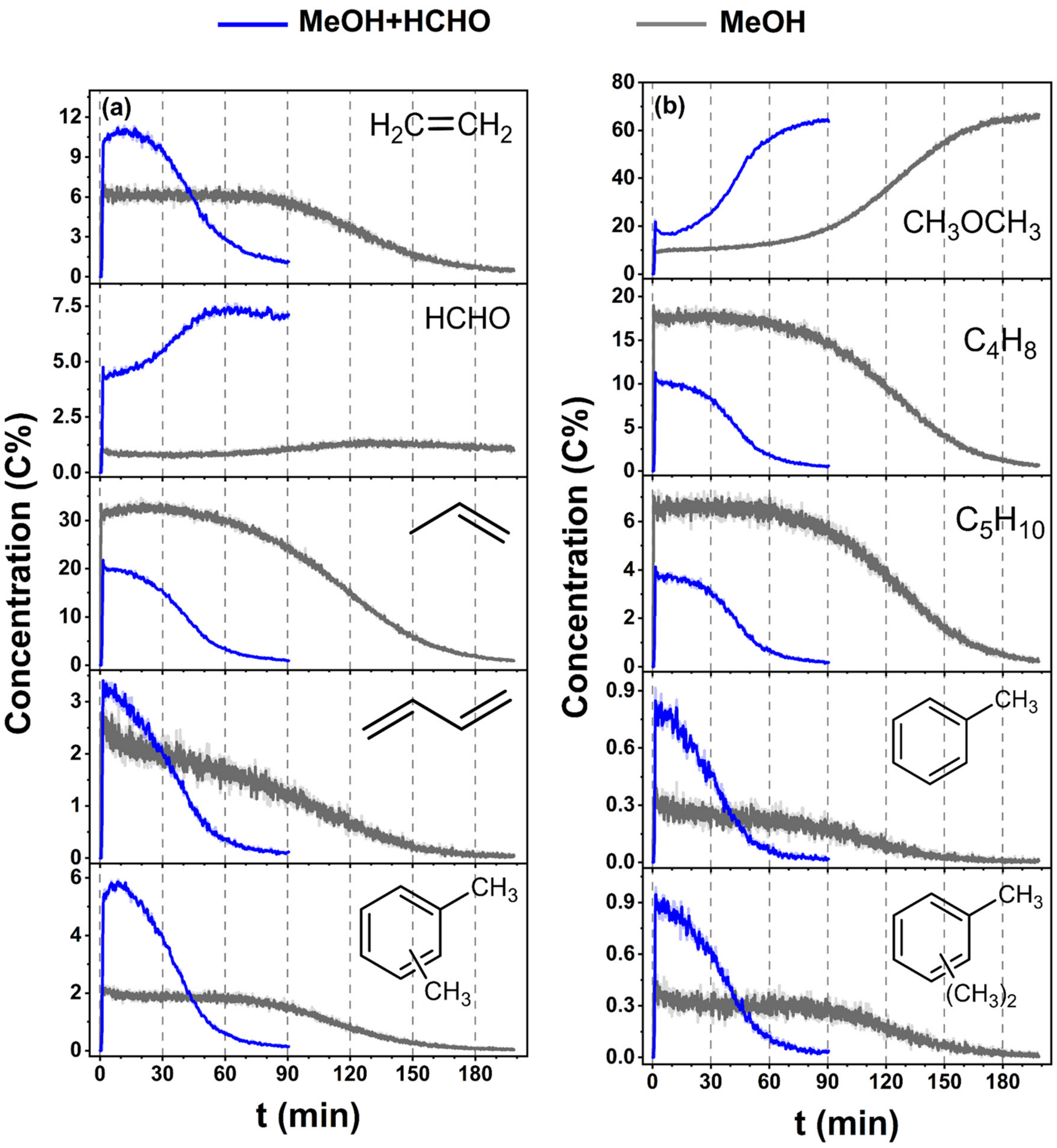

Supplementary Fig. 5. The real-time yields (C%) of (a) C_2H_4, HCHO, propylene, 1,3-butadiene, C8 aromatics, and (b) CH_3OCH_3, C_4H_8, C_5H_{10}, C_7H_8, C_9H_{12} in the MTH reaction with or without co-feeding 5 wt.% HCHO over parent HZSM-5. Reaction conditions: 400 °C; methanol WHSV = 12.52 $g_{MeOH}g_{catalyst}^{-1}h^{-1}$; 5 wt.% of trioxane mixed with methanol; P = 2 Torr; and each reaction proceeded until the ethylene yield dropped to 0.5 C%. The original yield curves (transparent solid lines) obtained in the experiments have been smoothed as bright solid lines. CH_3OCH_3 = dimethyl ether; C_4H_8 = $C_4^=$ olefins; C_5H_{10} = $C_5^=$ olefins; C_7H_8 = toluene; and C_9H_{12} = C_9 aromatics.

6. Supplementary graph in relationship between H_2/HCHO/hydrocarbon production and Ga content

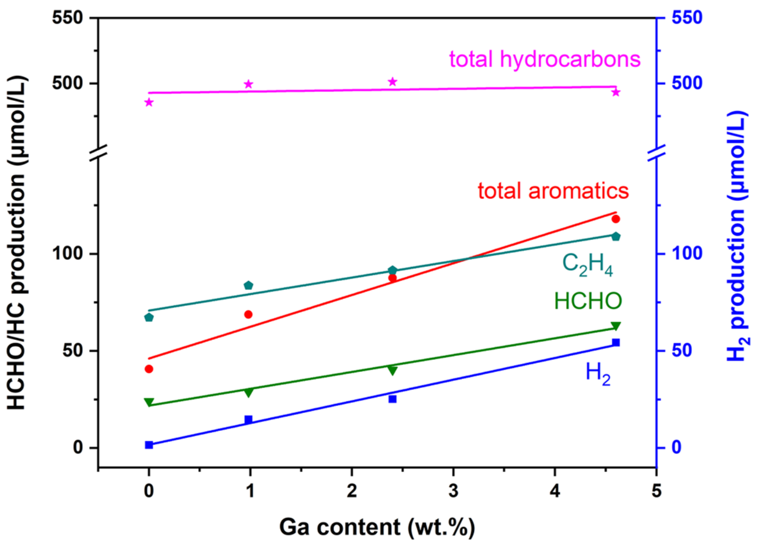

Supplementary Fig. 6. The mole concentrations of HCHO, C_2H_4, total aromatics, total hydrocarbons and H_2 versus Ga content. Total aromatics: the summation of C6-C9 aromatics in Table S2. Total hydrocarbon: the summation of all hydrocarbon products presented in Table S2. The mole concentrations of H_2 and C_2H_4 were calculated from their productions presented in Table S2. The mole concentration of HCHO was calculated from the yields of HCHO presented in Table S1. The concentrations of HCHO, C_2H_4, total aromatics and total hydrocarbons were calculated as the carbon atoms concentrations in corresponding products.

7. Supplementary graph of catalyst characterizations

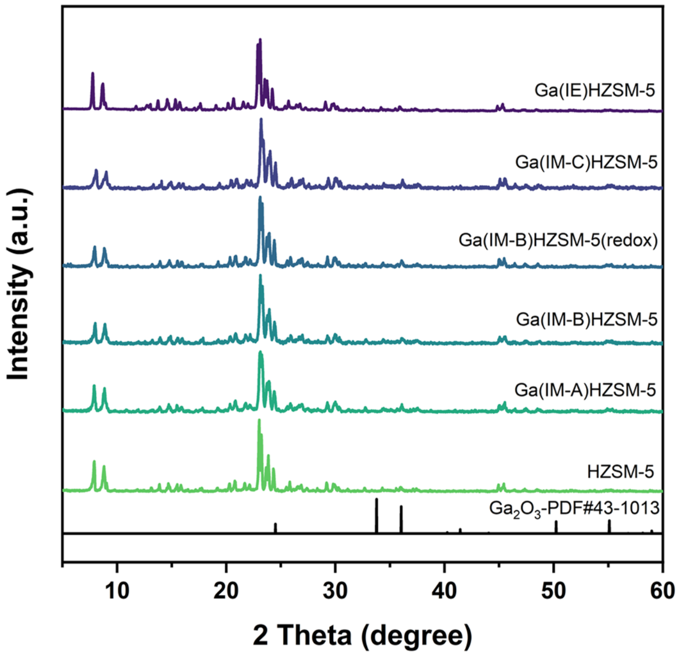

Supplementary Fig. 7. XRD patterns of parent HZSM-5 and Ga-modified HZSM-5.

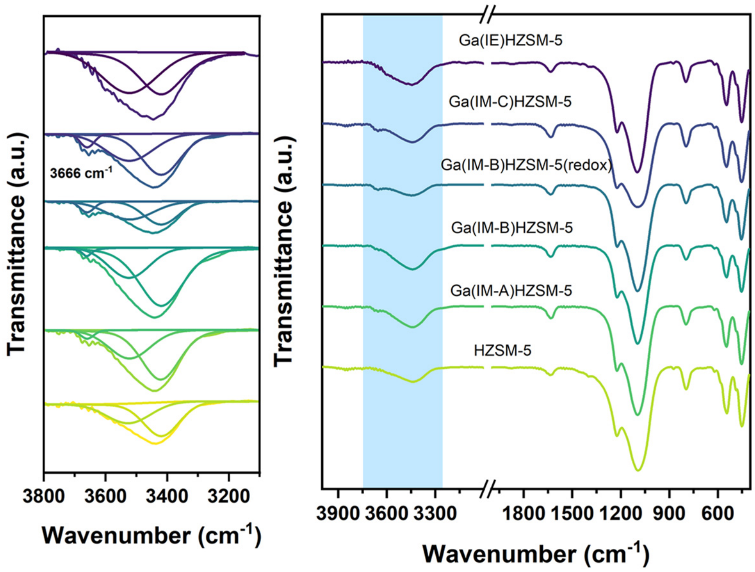

Supplementary Fig. 8. The FT-IR spectra of parent HZSM-5 and Ga-modified HZSM-5.

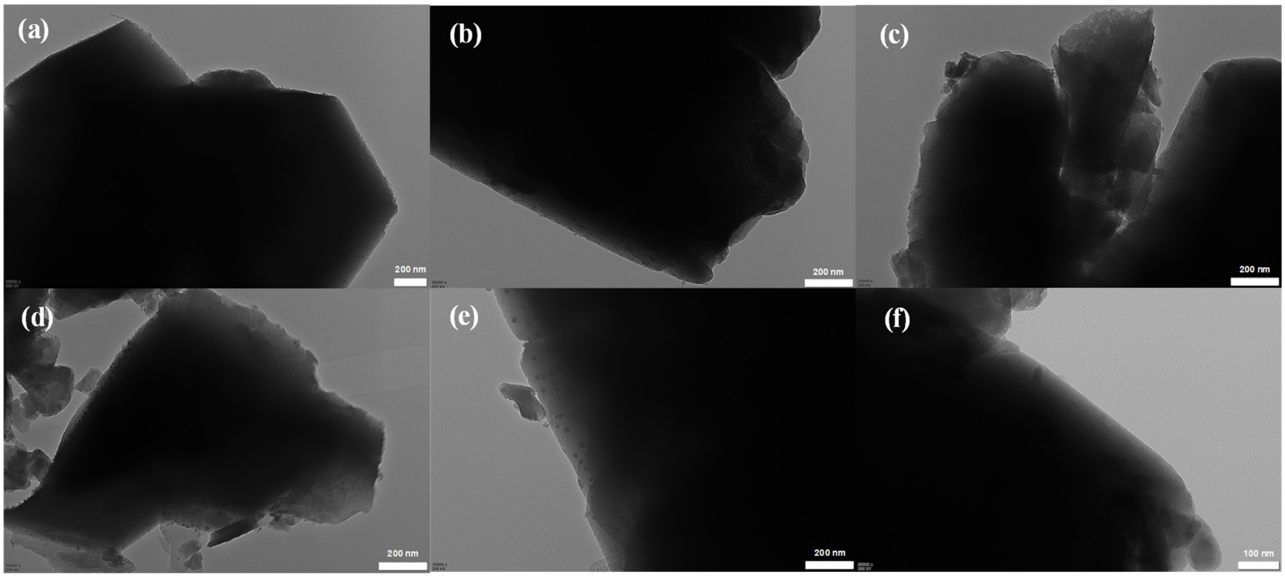

Supplementary Fig. 9. TEM images of (a) HZSM-5, (b) Ga(IM-A)HZSM-5, (c) Ga(IM-B)HZSM-5, (d) Ga(IM-B)HZSM-5(redox), (e) Ga(IM-C)HZSM-5, (f) Ga(IE)HZSM-5.

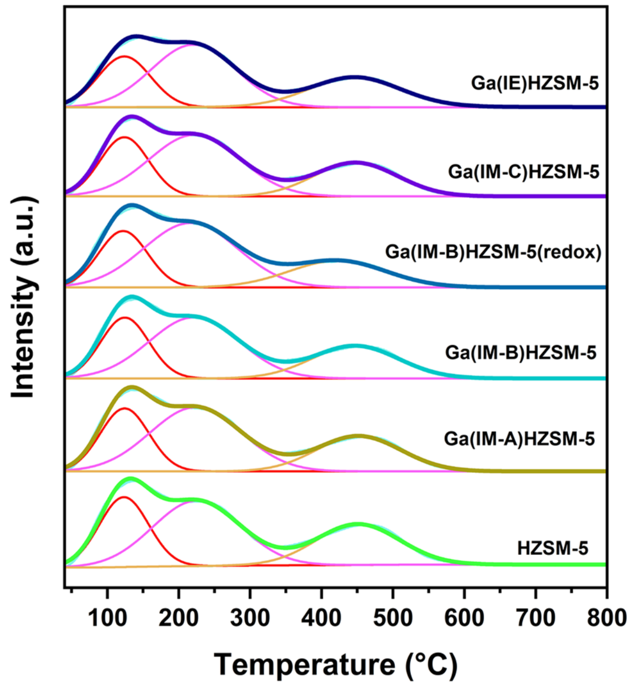

Supplementary Fig. 10. NH_3-TPD patterns of parent HZSM-5 and Ga-modified HZSM-5.

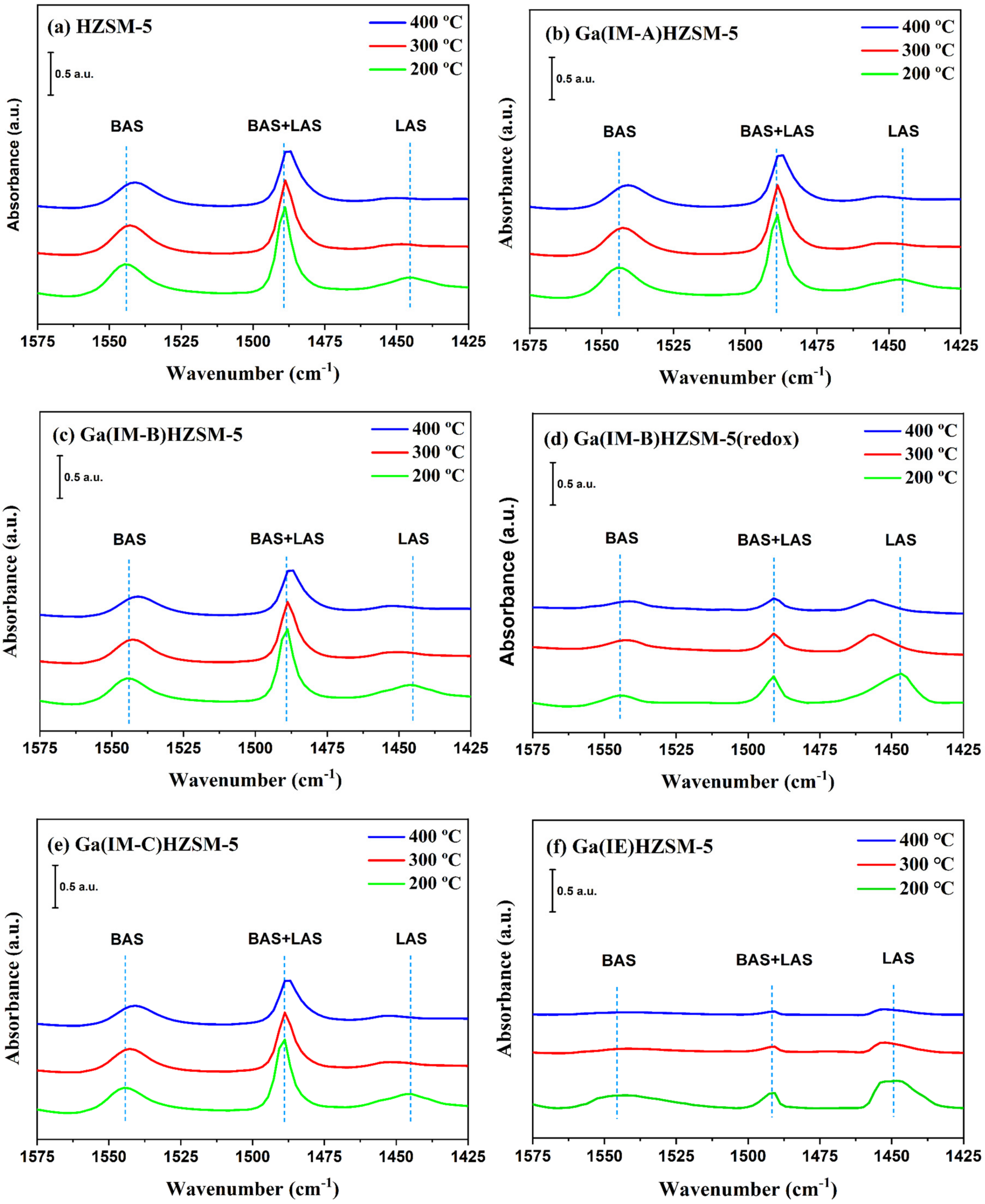

Supplementary Fig. 11. FT-IR spectrums of pyridine adsorbed on (a) HZSM-5, (b) Ga(IM-A)HZSM-5, (c) Ga(IM-B)HZSM-5, (d) Ga(IM-B)HZSM-5(redox), (e) Ga(IM-C)HZSM-5, (f) Ga(IE)HZSM-5.

8. Supplementary mass spectrum in MTH over Silicalite-1 and Ga-modified Silicalite-1

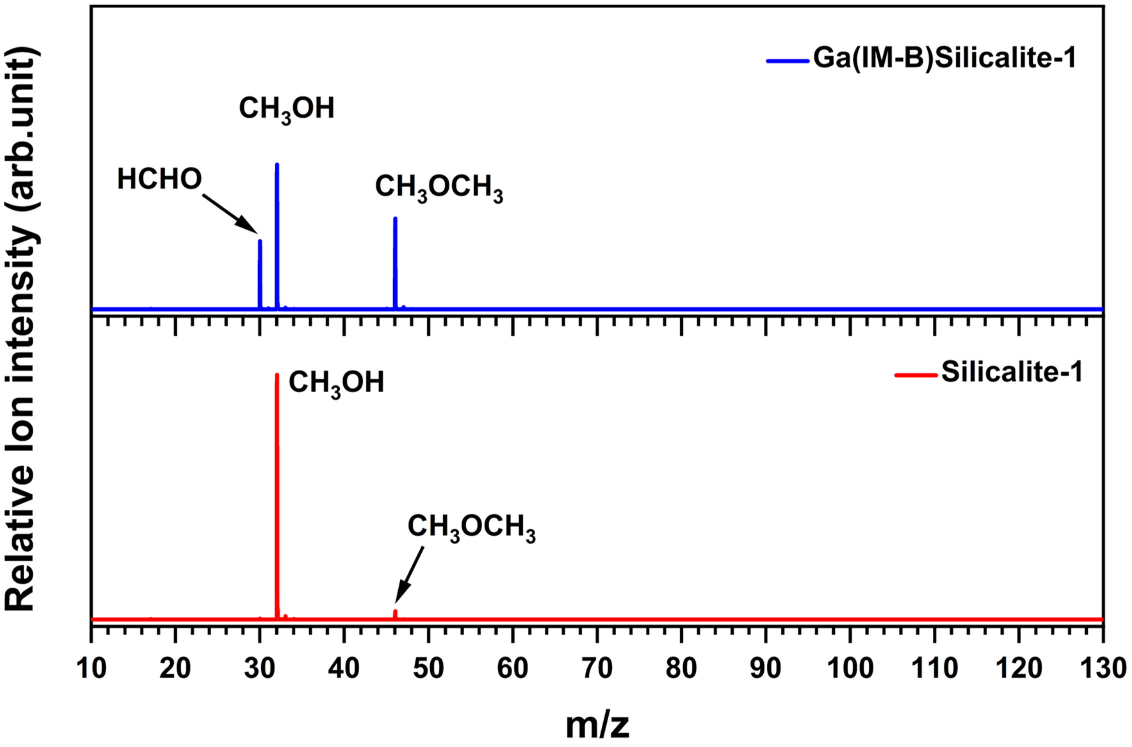

Supplementary Fig. 12. Photoionization mass spectrums of the products in MTH over Silicalite-1 and Ga(IM-B)Silicalite-1 at the photon energy of 11.0 eV. Reaction conditions of SR-PIMS experiments: 400 °C; methanol WHSV = 12.52 $g_{MeOH}g_{catalyst}^{-1}h^{-1}$; and P = 2 Torr.

9. Supplementary mass spectrum in MTH over single Ga_2O_3

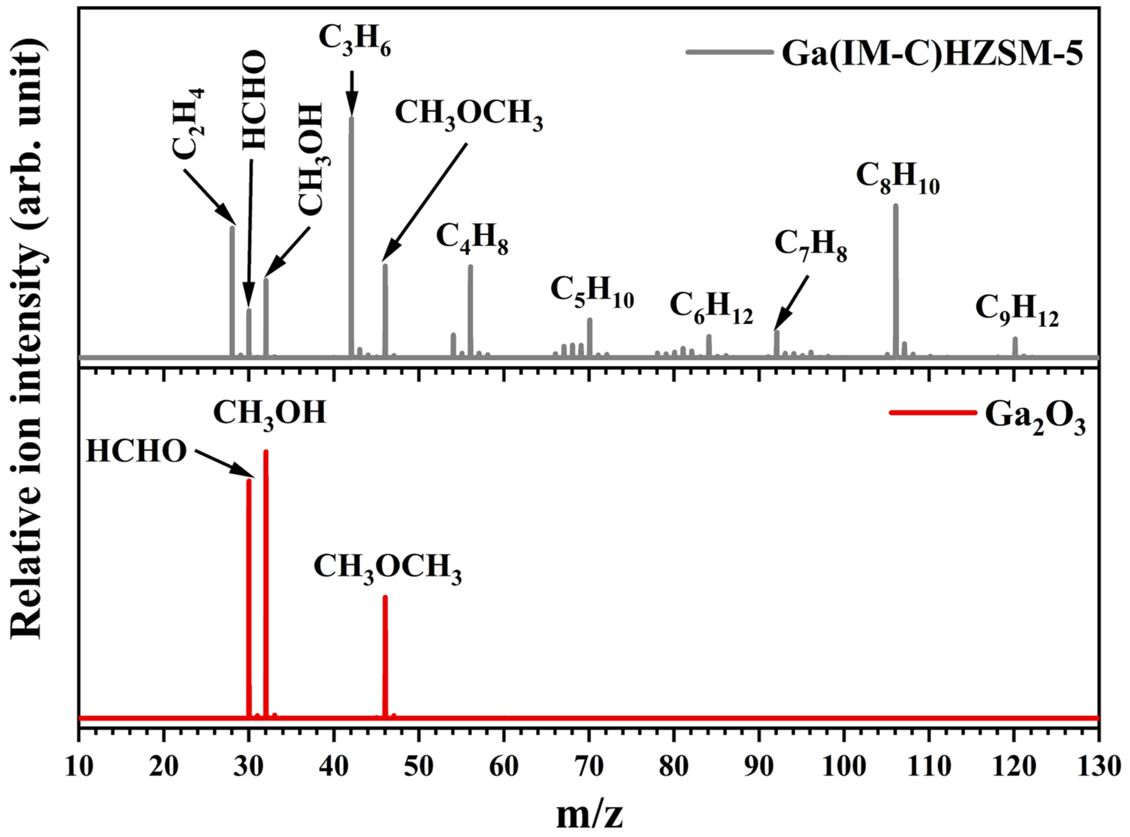

Supplementary Fig. 13. Photoionization mass spectrums of the products in MTH over Ga(IM-C)HZSM-5 and single Ga_2O_3 at the photon energy of 11.0 eV. Reaction conditions of SR-PIMS experiments: 400 °C; methanol WHSV = 12.52 $g_{MeOH}g_{catalyst}^{-1}h^{-1}$; and P = 2 Torr.

10. Supplementary graph in MTH over HZSM-5, Ga_2O_3/HZSM-5 and Ga_2O_3/HZSM-5/Y_2O_3

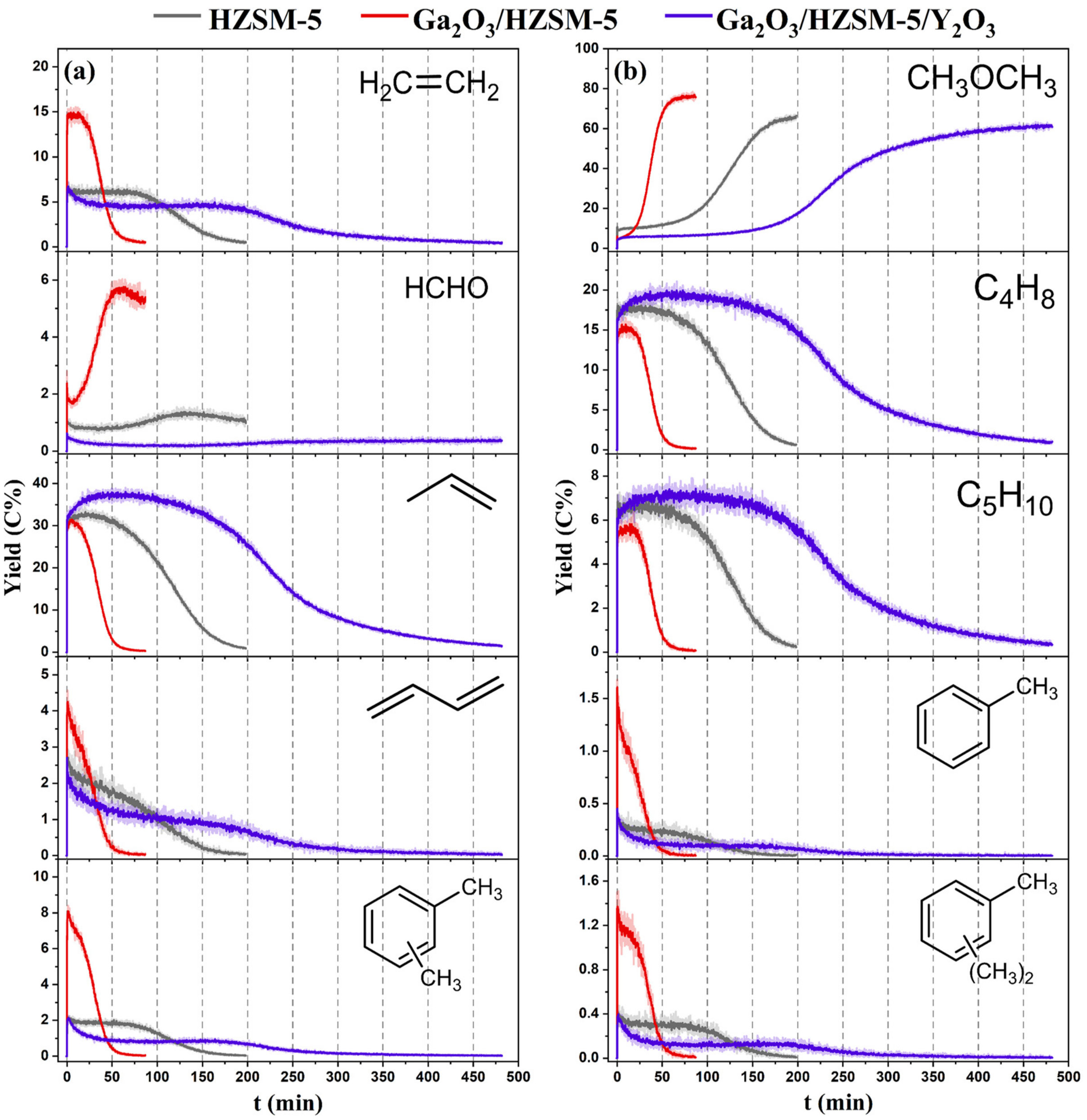

Supplementary Fig. 14. The real-time yields (C%) of (a) C_2H_4, HCHO, propylene, 1,3-butadiene, C_8 aromatics, and (b) CH_3OCH_3, C_4H_8, C_5H_{10}, C_7H_8, C_9H_{12} in the MTH reaction over parent HZSM-5, Ga_2O_3/HZSM-5 and Ga_2O_3/HZSM-5/Y_2O_3. Reaction conditions: 400 °C; methanol WHSV = 12.52 $g_{MeOH}g_{catalyst}^{-1}h^{-1}$; P = 2 Torr; and each reaction proceeded until the ethylene yield dropped to 0.5 C%. The original yield curves (transparent solid lines) obtained in the experiments have been smoothed as bright solid lines. CH_3OCH_3 = dimethyl ether; C_4H_8 = $C_4^=$ olefins; C_5H_{10} = $C_5^=$ olefins; C_7H_8 = toluene; and C_9H_{12} = C_9 aromatics.

11. Supplementary graph in MTH over HZSM-5, Ga(IE)HZSM-5 and Ga(IM-D)HZSM-5

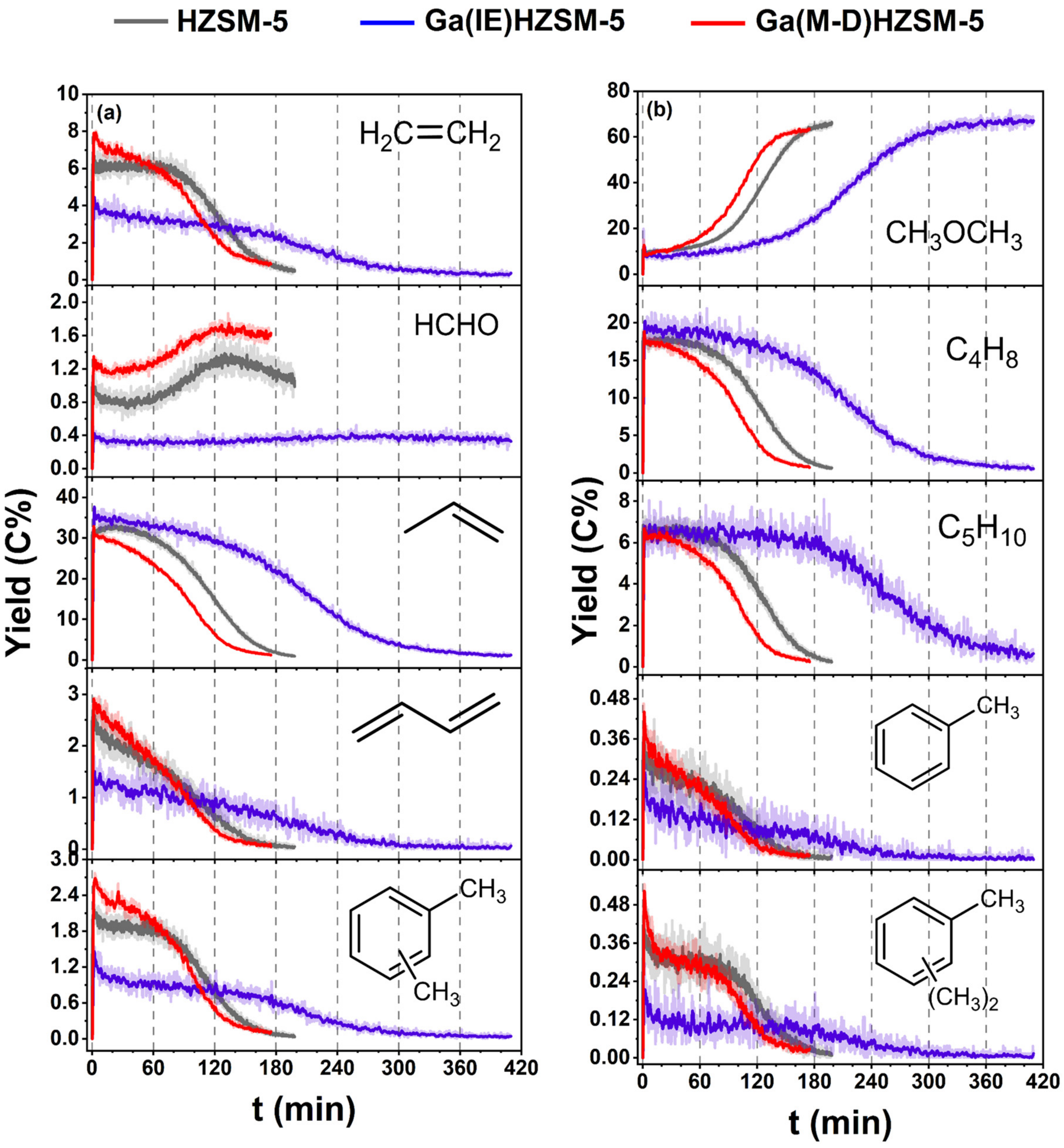

Supplementary Fig. 15. The real-time yields (C%) of (a) C_2H_4, HCHO, propylene, 1,3-butadiene, C8 aromatics, and (b) CH_3OCH_3, C_4H_8, C_5H_{10}, C_7H_8, C_9H_{12} in the MTH reaction over parent HZSM-5, Ga(IE)HZSM-5 and Ga(IM-D)HZSM-5. Reaction conditions: 400 °C; methanol WHSV = 12.52 $g_{MeOH}g_{catalyst}^{-1}h^{-1}$; P = 2 Torr; and each reaction proceeded until the ethylene yield dropped to 0.5 C%. The original yield curves (transparent solid lines) obtained in the experiments have been smoothed as bright solid lines. CH_3OCH_3 = dimethyl ether; C_4H_8 = C4= olefins; C_5H_{10} = C5= olefins; C_7H_8 = toluene; and C_9H_{12} = C9 aromatics.

12. Supplementary graph in MTH over HZSM-5, Ga(IM-B)HZSM-5 and Ga(IM-B)HZSM-5(redox)

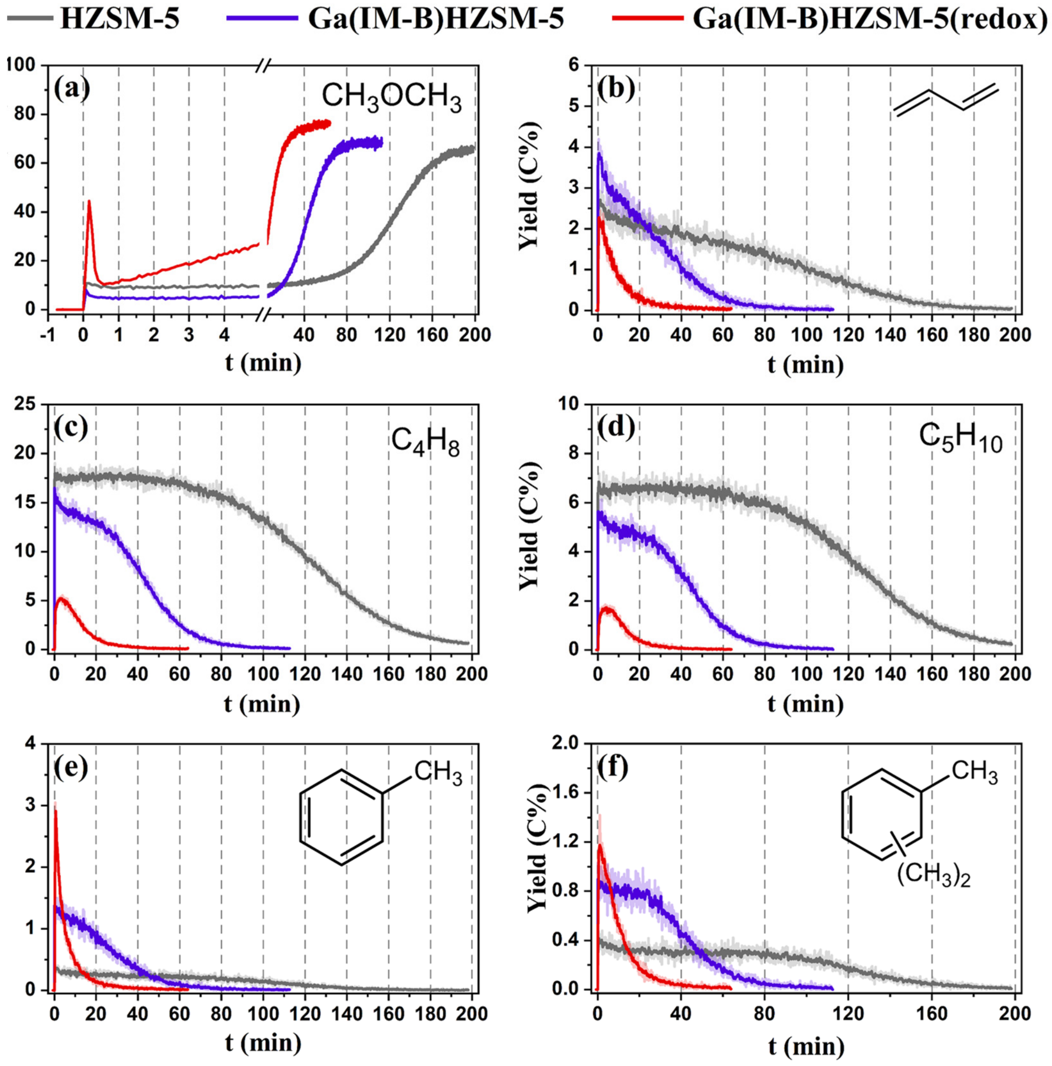

Supplementary Fig. 16. The real-time yields (C%) of (a) CH_3OCH_3, (b) 1,3-butadiene, (c) C_4H_8, (d) C_5H_{10}, (e) toluene and (f) C_9 aromatics in the MTH reaction over parent HZSM-5, Ga(IM-B)HZSM-5 and Ga(IM-B)HZSM-5(redox). Reaction conditions of SR-PIMS experiments: 400 °C; methanol WHSV = 12.52 $g_{MeOH}g_{catalyst}^{-1}h^{-1}$; P = 2 Torr; and each reaction proceeded until the ethylene yield dropped to 0.5 C%. The solid line in (a) was the original yield curve. In (b-e), the original yield curves (transparent solid lines) obtained in the experiments have been smoothed as bright solid lines. CH_3OCH_3 = dimethyl ether; C_4H_8 = $C_4^=$ olefins and C_5H_{10} = $C_5^=$ olefins.

13. Supplementary detailed reaction routes of aromatics formation

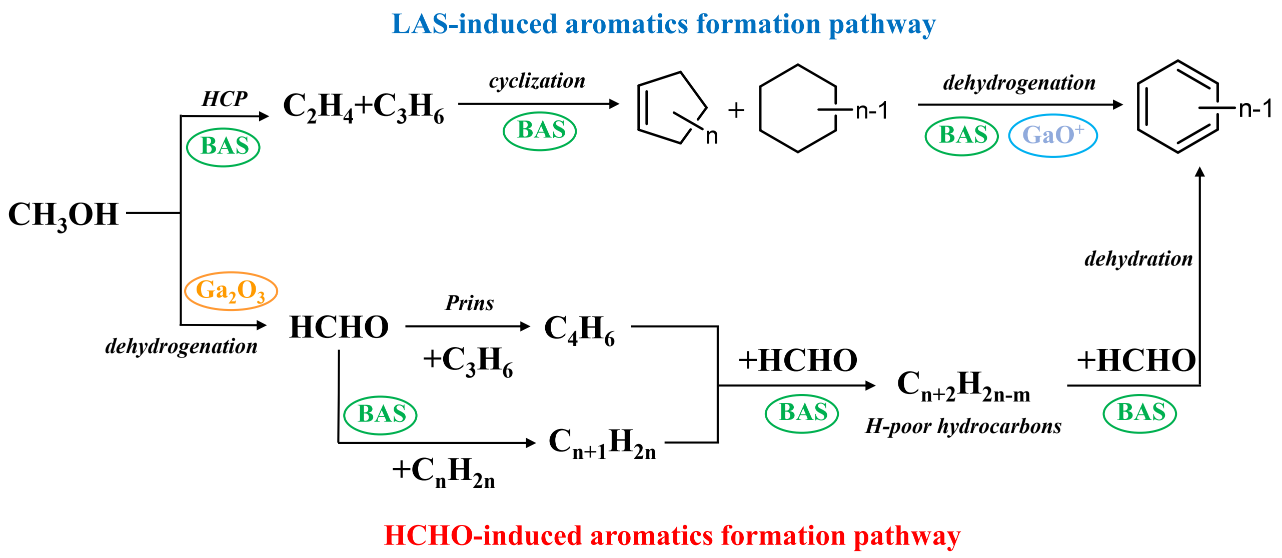

Supplementary Fig. 17. The details of HCHO-induced and LAS-induced aromatics formation pathway over various active centres.

14. Detailed results of MTH reaction under low pressure obtained by SR-PIMS experiments

Supplementary Table 1. The conversion and product yield of MTH reaction over parent HZSM-5 and Ga-modified HZSM-5 obtained by SR-PIMS experiments. [a]

Sample	HZSM-5	Ga(IM-A)HZSM-5	Ga(IM-B)HZSM-5	Ga(IM-C)HZSM-5
Conversion (%)	88.99	90.34	91.95	92.83
Product yield (C%)				
CH_4[b]	2.16	2.20	2.23	2.13
C_2H_4	6.18	11.26	14.15	17.10
HCHO	0.87	1.04	1.45	2.28
C_3H_6	32.68	31.00	30.78	26.96
CH_3OCH_3	10.00	8.23	5.89	5.62
C_4H_6	2.34	2.62	2.93	3.69
C_4H_8	17.62	14.45	14.37	10.90
C_5H_{10}	6.47	5.37	4.80	3.90
C_7H_8	0.26	0.75	1.04	1.75
C_8H_{10}	1.81	4.49	6.81	9.16
C_9H_{12}	0.36	0.56	0.98	1.10

[a] Reaction conditions: 400 °C; methanol WHSV = 12.52 $g_{MeOH}g_{catalyst}^{-1}h^{-1}$; and P = 2 Torr. These data were obtained during the steady-state reaction period of MTH reaction for each catalyst sample. C_3H_6 = propylene; CH_3OCH_3 = dimethyl ether; C_4H_6 = 1,3-butadiene; C_4H_8 = $C_4^=$ olefins; C_5H_{10} = $C_5^=$ olefins; C_7H_8 = toluene; C_8H_{10} = C_8 aromatics; and C_9H_{12} = C_9 aromatics. [b] Methane was detected at the photon energy of 14 eV, and other products were detected at 11 eV.

15. Detailed results of MTH reaction under atmospheric pressure obtained by GC-MS

Supplementary Table 2. The conversion, H_2 production and product yield of MTH reaction over parent HZSM-5, Ga-modified HZSM-5 and pure Ga_2O_3 obtained by GC-MS.[a]

Sample	HZSM-5	Ga(IM-A) HZSM-5	Ga(IM-B) HZSM-5	Ga(IM-C) HZSM-5	Ga(IM-A) HZSM-5/Y_2O_3	Ga_2O_3 /HZSM-5	Ga_2O_3 /HZSM-5/Y_2O_3	Ga(IM-B) HZSM-5(redox)	Ga(IE) HZSM-5	Ga_2O_3
Conversion (%)	100.00	100.00	100.00	100.00	100.00	100.00	100.00	100.00	100.00	65.56
H_2 production (μmol/L)	1.58	14.79	25.23	54.34	84.64	26.54	70.71	61.39	6.77	406.04
Product yield (C%)										
CO	0.00	0.00	0.00	0.00	7.58	0.00	5.81	0.00	0.00	3.87
CO_2	0.00	0.00	0.00	0.00	0.00	0.00	0.00	0.00	0.00	9.63
CH_4	0.92	1.21	1.26	1.33	1.02	1.19	0.88	1.12	1.09	0.26
C_2H_4	12.49	15.55	17.01	20.24	13.13	14.78	11.48	22.21	20.98	0.00
C_2H_6	0.00	0.00	0.00	0.00	0.00	0.00	0.00	1.71	0.14	0.00
C_3H_6	42.96	37.26	35.02	30.72	39.3	37.05	41.41	11.16	30.21	0.00
C_3H_8	1.93	2.25	2.32	1.86	2.30	1.66	1.91	1.30	3.47	0.00
CH_3OCH_3	0.00	0.00	0.00	0.00	0.00	0.00	0.00	0.00	0.00	22.06
C_4H_8	16.04	13.37	12.64	10.23	15.11	13.62	16.03	2.81	10.01	0.00
C_4H_{10}	3.91	4.49	4.04	1.83	4.95	2.36	4.01	0.85	4.50	0.61
C_5H_{10}	3.27	2.81	2.80	2.56	2.40	3.80	2.85	0.36	4.48	0.00
C_5H_{12}	1.97	2.41	1.84	1.04	2.59	1.17	2.17	0.31	2.50	0.00
C_6H_6	0.27	0.49	0.63	1.00	0.38	0.47	0.23	3.12	0.67	0.00
C_7H_8	1.77	3.84	3.85	5.36	1.68	3.85	1.78	8.83	4.29	0.00
C_8H_{10}	4.01	8.36	10.64	14.47	3.99	8.99	4.06	17.08	9.51	0.00
C_9H_{12}	0.78	0.84	1.17	1.11	0.29	1.13	0.49	1.33	1.17	0.00
Total aromatics	6.83	13.53	16.29	21.94	6.3 4	14.44	6.56	30.36	15.64	0.00

[a] Reaction conditions: 400 °C; methanol WHSV = 1.75 $g_{MeOH}g_{catalyst}^{-1}h^{-1}$; and pressure was atmospheric pressure. These data were obtained during the steady-state reaction period of MTH reaction for each catalyst sample. C_3H_6 = propylene; CH_3OCH_3 = dimethyl ether; C_4H_8 = $C_4^=$ olefins; C_4H_{10} = C_4 alkanes; C_5H_{10} = $C_5^=$ olefins; C_5H_{12} = C_5 alkanes; C_6H_6 = benzene; C_7H_8 = toluene; C_8H_{10} = C_8 aromatics; C_9H_{12} = C_9 aromatics; and total aromatics are the summation of C6-C9 aromatics yield.

16. Supplementary references

1 Zhou, Z. Y. *et al.* The vacuum ultraviolet beamline/endstations at NSRL dedicated to combustion research. *J. Synchrotron Radiat.* **23**, 1035-1045, (2016).

2 Wen, W. *et al.* Formation and fate of formaldehyde in methanol-to-hydrocarbon reaction: in situ synchrotron radiation photoionization mass spectrometry study. *Angew. Chem. Int. Ed. Engl.* **59**, 4873-4878, (2020).

3 Cool, T. A. *et al.* Photoionization mass spectrometer for studies of flame chemistry with a synchrotron light source. *Rev. Sci. Instrum.* **76**, (2005).

4 Qi, F. & McIlroy, A. Identifying combustion intermediates via tunable vacuum ultraviolet photoionization mass spectrometry. *Combust. Sci. Technol.* **177**, 2021-2037, (2005).

5 Liu, Y. *et al.* Formation mechanism of the first carbon-carbon bond and the first olefin in the methanol conversion into hydrocarbons. *Angew. Chem. Int. Ed. Engl.* **55**, 5723-5726, (2016).

6 Chowdhury, A. D. *et al.* Initial carbon-carbon bond formation during the early stages of the methanol-to-olefin process proven by zeolite-trapped acetate and methyl acetate. *Angew. Chem. Int. Ed. Engl.* **55**, 15840-15845, (2016).

7 Qi, L., Wei, Y. X., Xu, L. & Liu, Z. M. Reaction behaviors and kinetics during induction period of methanol conversion on HZSM-5 zeolite. *ACS Catal.* **5**, 3973-3982, (2015).

8 Olsbye, U. *et al.* Conversion of methanol to hydrocarbons: how zeolite cavity and pore size controls product selectivity. *Angew. Chem. Int. Ed. Engl.* **51**, 5810-5831, (2012).

9 Goetze, J. *et al.* Insights into the activity and deactivation of the methanol-to-olefins process over different small-pore zeolites as studied with operando UV-vis spectroscopy. *ACS Catal.* **7**, 4033-4046, (2017).
