# Supplementary material for: Role of formaldehyde in promoting aromatic selectivity during methanol conversion over gallium-modified zeolites
Source: Commun Chem. 2022 Nov 19;5:153. doi: 10.1038/s42004-022-00771-8 (PMC9814038; doi:10.1038/s42004-022-00771-8)
Supplement: Supplementary file 1 — Pan_PR File [file 42004_2022_771_MOESM1_ESM.pdf]

nature portfolio

Peer Review File

Role of formaldehyde in promoting aromatic selectivity during methanol conversion over gallium-modified zeolites

Open Access This file is licensed under a Creative Commons Attribution 4.0 International License, which permits use, sharing, adaptation, distribution and reproduction in any medium or format, as long as you give appropriate credit to the original author(s) and the source, provide a link to the Creative Commons license, and indicate if changes were made. In the cases where the authors are anonymous, such as is the case for the reports of anonymous peer reviewers, author attribution should be to 'Anonymous Referee' followed by a clear attribution to the source work. The images or other third party material in this file are included in the article's Creative Commons license, unless indicated otherwise in a credit line to the material. If material is not included in the article's Creative Commons license and your intended use is not permitted by statutory regulation or exceeds the permitted use, you will need to obtain permission directly from the copyright holder. To view a copy of this license, visit http://creativecommons.org/licenses/by/4.0/.

Reviewers' comments:

Reviewer #1 (Remarks to the Author):

In this work, the causation of Ga Species in enhancing the aromatics production over Ga-modified H-ZSM-5 zeolites for methanol conversion was investigated. It was clarified that Ga2O3 in Ga-modified H-ZSM-5, obtained by impregnation, was catalytically active for the formation of formaldehyde during methanol conversion and formaldehyde produced over Ga2O3 in the reaction mixture could promote the production of aromatics.

Such a result is interesting and may evoke further investigation on the related subject. It deserves publication; however, this reviewer knows little about the new journal of Communications Chemistry and has no ideas about whether it accords with the readership as well as the academic request in novelty and research depth.

Nevertheless, there are considerable uncertainties which should be considered before it can be published.

(1) It seems that the promoting effect of Ga modification on methanol conversion to aromatics is ascribed to the capacity of Ga2O3 in boosting the formation of formaldehyde. In this regard, a comparative test by co-feeding methanol and proper quantity of formaldehyde over H-ZSM-5 should be provided.

(2) Various catalysts like single Ga2O3, H-ZSM-5, Ga(IM)/HZSM-5, Ga2O3/HZSM-5, etc. were compared. However, this reviewer suggests that similar to Ga(IM)/HZSM-5, a Ga(IM)/Silicalite-1 should be prepared, which have a similar structural properties as well as possible similar nature for the supported Ga species but little acidity. The performance of Ga(IM)/Silicalite-1 in formaldehyde formation and methanol conversion may give more proofs about the role of Ga species played in methanol conversion.

(3) It seems that formaldehyde may only have a significant promoting effect on the methanol conversion in the initial period. In the later periods, formaldehyde may even show negative effect on methanol conversion. More explanation and discussion should be provided in this regard.

(4) In the introduction, line 52, it was said “the synergic effect derived from the close spatial proximity between cationic Ga species and BASs leads to an enhanced Brønsted acidity”, what does the enhanced acidity mean here? A higher density or stronger acid sites?

(5) It is somewhat unfair to compare Ga(IM)/HZSM-5 and Ga(IE)/HZSM-5, when two catalysts are greatly different in the loading of Ga. In addition, Ga(IM)/HZSM-5 should also have considerable ion-exchanged Ga species.

(6) Is it possible that certain Ga is incorporated into the framework of MFI, especially after rigorous calcination treatments?

(7) Some grammatical issues like “the usage amounts of HZSM-5, Ga(IM)/HZSM-5, single Ga2O3 and Ga(IM)HZSM-5(redox) were 150 mg respectively”; etc.

Reviewer #2 (Remarks to the Author):

The manuscript "New Insights into the Role of Formaldehyde Generated over Ga Species in Promoting Aromatics in Methanol Conversion" describes a piece of research to unravel the role gallium modified

ZSM-5 in the MTG reaction. Utilising state of the art SR-PIMS analysis combined with separate online catalytic tests, the authors have shown that Ga2O3 species are responsible for the production of formaldehye, through the direct dehydrogenation of methanol, which results in elevated aromatics production. The findings will be of great interest to the very large MTG community, to the wider catalysis community, and also of interest to those working in the area of valorisation of feedstocks such as coal, gas and biomass.

My recommendation, based on the specific comments below, is:
Invite the authors to revise their manuscript to address specific concerns before a final decision is reached.

Scientifc comments:
The following scientific points are the main reason to ask for a revised manuscript to be submitted. The data produced by the SR-PIMS technique is very powerful, however the characterisation data of the materials made is not well described, and the experimental detail insufficient to be repeated by others.

The TEM images (Fig S7) are said to show that IE material does not contain Ga2O3 clusters, while the IM material has uniformly distributed clusters. This is not supported by the TEM images as it is only possible to discern particles around the very edge of the crystallites due to the poor TEM resolution (it looks as though the body of the crystallites are too thick for the e-beam to penetrate the material).

The lack of detail around band deconvolution for NH3 TPD is concerning given that it is used to characterise the different species present.

The authors offer no suggestion to the other deconvoluted bands in the FTIR spectrum at approx 3400 and 3500 cm-1. It is also surprising that there are no terminal silanol species at approximately 3700 cm-1. Can the authors explain why this band is absent?

Missing experimental detail (not exhaustive):
1) Where did the Ga and Y salts originate? Where appropriate, what grade of material was used? (e.g. methanol, Ga and Y sources, "pure hydrogen", air (synthetic or just from the lab?)?
2) Missing experimental details. What ramp rate was used for all heating protocols? How was the solid dissolved for ICP analysis? How were the TEM samples prepared? WHat were the degas conditions for BET analysis. Over what p/p0 range was applied for BET analysis? What method of FTIR was employed - transmission, DRIFTS, ATR? Were thermal treatments applied to the zeolite/KBr tablet prior to analysis? During pyridine desorption at elevated T, what duration was each T held at? What extinction coefficient was applied for LAS and BAS sites for quantitative determination? What is the uncertainty in the quantitative determination of BAS and LAS sites by pyridine adsorption? Were the catalysts tested as powders or pressed and sieved to a certain particle fraction?
3) Please could the authors add the Ga/Al ratios of the materials to Table 1.

General comments:
The text needs careful revision to ensure that the scientific message is clear and can be understood by readers. I had to read the manuscript several times in order to understand the message. (e.g. sentence over lines 183-186, sentences over 227-231).
The authors use language which is overly confident in my opinion: (144-146: "the lack of GaOH peak in FT-IR spectra and Ga2O3 particles in TEM for Ga(IE)HZSM-5 demonstrated that only cationic GaO+ was left after" - there could be other explanation's, e.g. below limit of detection; 178-180 "In other word, Ga2O3 could be uncontroversially assigned as the active phase in Ga(IM)HZSM-5 for the dehydrogenation of MeOH to HCHO" - "strongly indicates" would be better than "uncontroversially").
It would help readers if a line summing the aromatics product yield was added to Table S2.
Line 206-208 should make reference to Table 1.

The manuscript line 319 states that the HayeSep column was used for inorganic analysis - surely this was a typographical error? CO_2 is not an inorganic molecule.

Reviewer #3 (Remarks to the Author):

In this contribution, Yang Pan and co-workers use synchrotron-based photo-ionisation mass spectrometry to study the formaldehyde-induced conversion of methanol to aromatics (and other products) over H-ZSM-5 catalysts, either as such, or modified/ mixed with Ga^{3+}/Ga_2O_3 and potentially Y_2O_3. The study is a follow-up of a prior contribution from the same group, where they studies ZSM-5 with and without addition of Y_2O_3 by using the same method.
In this contribution, the authors observe that aromatics formation is correlated with H_2 formation, and conclude that CH_2O formation may proceed over two different Ga sites: Ga ion-exchanged onto the Brønsted acid site of the zeolite, and Ga_2O_3 inside or outside the ZSM-5 crystals.
The study is novel and reports interesting observations that will most certainly be appreciated by the catalysis community. I suggest acceptance after properly taking into account the following comments:
1. The correlation of formaldehyde formation to hydrogen formation is based on their concomitant increase with different material combination, and I appreciate that. However, there is no direct comparison between the molar amount of H_2 and HCHO/ HC products formed in the experiments. This information should be provided in the revised manuscript. In our own research, we found that the amount of H_2 formed in similar (yet less advanced) experiments was always lower than expected, and we have not sorted out why. In case the authors made a similar observation, a discussion of this issue would be appreciated.
2. As shown by this study and the authors' prior study, as well as literature studies, formaldehyde is a precursor to aromatics formation. Moreover, according to the latest mechanistic studies of the initiation reaction, i.e. the formation of the first C-C bond in MTH, formaldehyde could either react directly with methanol/methoxy, or split to CO and H_2 followed by carbonylation of a methoxy species, to form the first C-C bond. In light of those literature studies, it is of interest to know what happens to the initiation rate when adding Ga and when adding Ga + Y_2O_3, i.e. to what extent does the formaldehyde formation affect the rate of formation of the first HC species? Please report and comment.
3. On page 5, the authors state: "Due to the formation of ethylene is closely associated with the aromatics-based cycle…, the yield of ethylene increased obviously with the enhanced production of aromatics." This statement may be correct, but I find it counterintuitive to correlate ethylene formation only to the aromatics cycle as long as ethylene appears before the aromatic product in Figure 3 e. Are there other aromatic products formed before ethylene, which led to the authors' statement? Did the time before appearance of each product change between materials? Please comment.
4. For the readers' information, please add CH_4 to Fig. 3e.
5. Reproducibility. To what extent have the reported results and trends between materials been confirmed by reproducibility tests? Please report and add reproducibility results with error bars to the manuscript.
6. Scheme 1 is too schematic. Please draw, either in main manuscript or in SI, the suggested mechanism of formation of each product, including a distinction between the roles of BAS and LAS in each reaction.
7. In the prior contribution on ZSM-5 with and without Y_2O_3 addition, a direct correlation was observed between methane and formaldehyde formation over ZSM-5. Here, that is not the case. It would be interesting to know what the authors think of this difference – is the CH_4-based route completely suppressed in the presence of Ga? Please elaborate.

Minor comment:
8. The manuscript needs some language improvement before publication. Much of it is well written, but then there are sentences / statements that need correction, such as the following examples:

In Abstract, line 2: “However, the active sites … are still debating.”
Page 3, 6th last line: “It was suggested that there may exist some certain active sites…”
Page 3, 3rd last line: “…, which has aroused wide interest from more and more investigators recently.”
Page 6, 4th last line: “… Rxn 1 and Rxn 2 could not interpret the increment of HCHO.”

We truly appreciate the reviewers for their very professional and detailed suggestions which help us to improve this work. Revisions have been made throughout the manuscript following the suggestions from the reviewers. The point-to-point responses to the comments by Reviewers are given below:

Reviewer #1:
In this work, the causation of Ga Species in enhancing the aromatics production over Ga-modified H-ZSM-5 zeolites for methanol conversion was investigated. It was clarified that Ga_2O_3 in Ga-modified H-ZSM-5, obtained by impregnation, was catalytically active for the formation of formaldehyde during methanol conversion and formaldehyde produced over Ga_2O_3 in the reaction mixture could promote the production of aromatics.

Such a result is interesting and may evoke further investigation on the related subject. It deserves publication; however, this reviewer knows little about the new journal of Communications Chemistry and has no ideas about whether it accords with the readership as well as the academic request in novelty and research depth.

Nevertheless, there are considerable uncertainties which should be considered before it can be published.

(1) It seems that the promoting effect of Ga modification on methanol conversion to aromatics is ascribed to the capacity of Ga_2O_3 in boosting the formation of formaldehyde. In this regard, a comparative test by co-feeding methanol and proper quantity of formaldehyde over H-ZSM-5 should be provided.

Response: We appreciate your suggestion. We have added the comparative test by co-feeding methanol and 5 wt.% HCHO over HZSM-5 (Figure R1). The significant increases in yields of aromatics and C_2H_4 by co-feeding HCHO confirmed the HCHO-involved aromatics formation pathway. Thus, it is reasonable to attribute the promoting effect of Ga-modified HZSM-5 on MTA to the capacity of Ga_2O_3 in boosting the formation of formaldehyde. This result was added in SI as Supplementary Fig. 5.

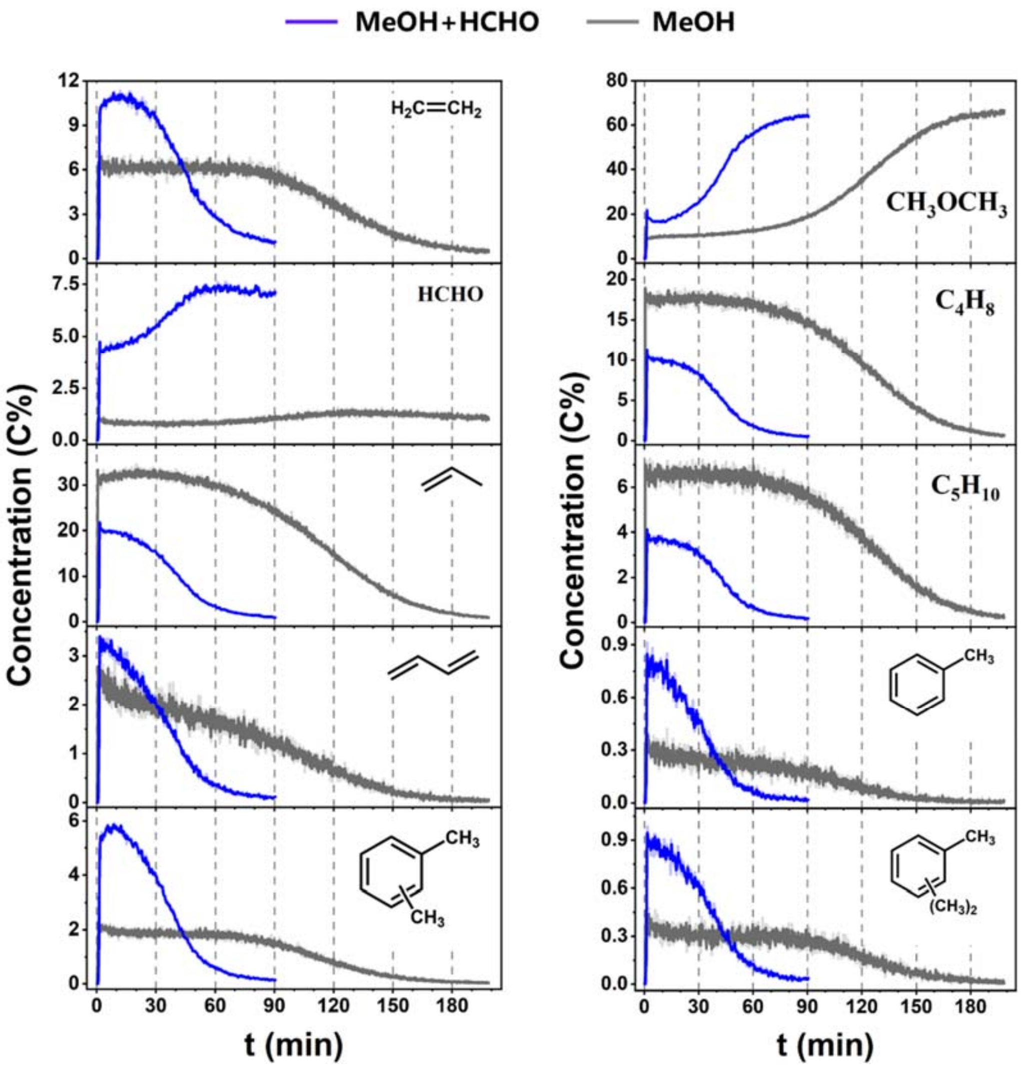

Figure R1. The real-time yields (C%) of (a) C_2H_4, HCHO, propylene, 1,3-butadiene, C8 aromatics, and (b) CH_3OCH_3, C_4H_8, C_5H_{10}, C_7H_8, C_9H_{12} in the MTH reaction with or without co-feeding 5 wt.% HCHO over parent HZSM-5. Reaction conditions: 400 °C; methanol WHSV = 12.52 $g_{MeOH}g_{catalyst}^{-1}h^{-1}$; 5 wt.% of trioxane mixed with methanol; P = 2 Torr; and each reaction proceeded until the ethylene yield dropped to 0.5 C%. The original yield curves (transparent solid lines) obtained in the experiments have been smoothed as bright solid lines. C H_3OCH_3 = dimethyl ether; C_4H_8 = $C_4^{=}$ olefins; C_5H_{10} = $C_5^{=}$ olefins; C_7H_8 = toluene; and C_9H_{12} = C_9 aromatics.

(2) Various catalysts like single Ga_2O_3, H-ZSM-5, Ga(IM)/HZSM-5, Ga_2O_3/HZSM-5, etc. were compared. However, this reviewer suggests that similar to Ga(IM)/HZSM-5, a Ga(IM)/Silicalite-1 should be prepared, which have a similar structural properties as well as possible similar nature for the supported Ga species but little acidity. The performance of Ga(IM)/Silicalite-1 in formaldehyde formation and methanol conversion may give more proofs about the role of Ga species played in methanol conversion.

Response: We appreciate your suggestion. We have supplemented the comparative tests of Silicalite-1 and Ga(IM-B)Silicalite-1 under typical reaction conditions of SR-PIMS experiments (Figure R2). It

obtained low conversion of methanol and small amount of DME over Silicalite-1 due to little acidity. After impregnating by 2.5 wt.% of Ga (Ga(IM-B)Silicalite-1), the conversion of methanol was promoted accompanying by increasing in formaldehyde and DME generation. The Ga_2O_3 dispersed on Silicalite-1 boosted the dehydrogenation of methanol to HCHO, and the new acid sites that originated from Ga species promoted the dehydration of methanol to DME. None of hydrocarbon products were detected, indicating that Brønsted acid sites are indispensable to MTH process. This result was added in SI as Supplementary Fig. 12.

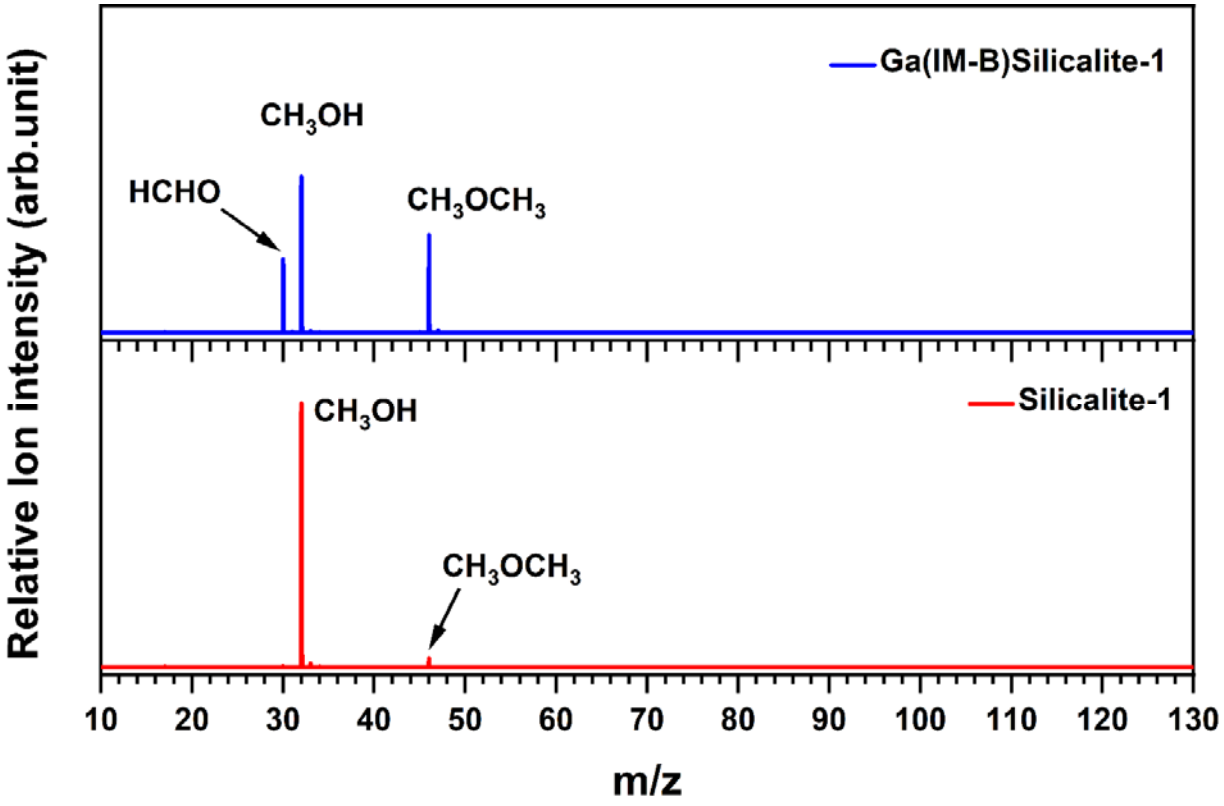

Figure R2. Photoionization mass spectrums of the products in MTH over Silicalite-1 and Ga(IM-B)Silicalite-1 at the photon energy of 11.0 eV. Reaction conditions of SR-PIMS experiments: 400 °C; methanol WHSV = 12.52 $g_{MeOH}g_{catalyst}^{-1}h^{-1}$; and P = 2 Torr.

(3) It seems that formaldehyde may only have a significant promoting effect on the methanol conversion in the initial period. In the later periods, formaldehyde may even show negative effect on methanol conversion. More explanation and discussion should be provided in this regard.

Response: Thanks for your question. Formaldehyde does have a significant promoting effect on the methanol conversion to aromatics, especially in the initial period. It is ascribed to the enhanced HCHO-induced aromatics formation pathway. However, the increasing of aromatics production accelerates catalyst deactivation owing to the severe coke deposit in the zeolite channel. Thus, methanol conversion is suppressed in the later period. It is indeed a huge challenge between the promoting aromatics formation and the decelerating catalyst deactivation.

(4) In the introduction, line 52, it was said "the synergic effect derived from the close spatial proximity between cationic Ga species and BASs leads to an enhanced Brønsted acidity", what does the enhanced acidity mean here? A higher density or stronger acid sites?

Response: We appreciate your question. This statement comes from the work of Deng and co-workers.[1] The influence of the spatial proximity/interaction between Ga species and BAS on the acidic property of Ga-modified HZSM-5 zeolites was explored via the ^{1}H MAS NMR spectra of [D_5]pyridine adsorption in their work. [D_5]pyridine is a well-established NMR probe to measure the Brønsted acid strength of zeolites. As shown in the Figure R3, the poorly resolved signals, ranging from 1.9 ppm to 4.3 ppm, correspond to SiOH, AlOH, and a small amount of BAS, which are inaccessible to pyridine molecules; the 8.7 and 8.1 ppm signals can be attributed to the formation of hydrogen bonds between pyridine and nonacidic SiOH; the low-field main signals at 15.6 and 19 ppm are due to the protonated pyridine on BAS. A new shoulder peak at 13.1 ppm appears in the ^{1}H MAS spectra of [D_5]pyridine adsorbed on Ga/ZSM-5(IM) and Ga/ZSM-5(redox), but it is absent in those of H-ZSM-5 and Ga_2O_3/ZSM-5. For [D_5]pyridine adsorbed on the BAS of zeolites, pyridine ion complexes are usually formed with ^{1}H chemical shifts in the range of 12–20 ppm, and a smaller ^{1}H chemical shift indicates a stronger Brønsted acid strength. Therefore, the observation of the 13.1 ppm signal demonstrates that the acid strength of some BAS is enhanced considerably on Ga/ZSM-5(IM) and Ga/ZSM-5(redox), because of the synergic effect of BAS and Ga species, which are generated by their spatial proximity/interaction. Thus, the enhanced acidity here means a stronger Brønsted acid strength. We have cited this literature in our work, reference 7. The reviewer can obtain more details from the original literature.

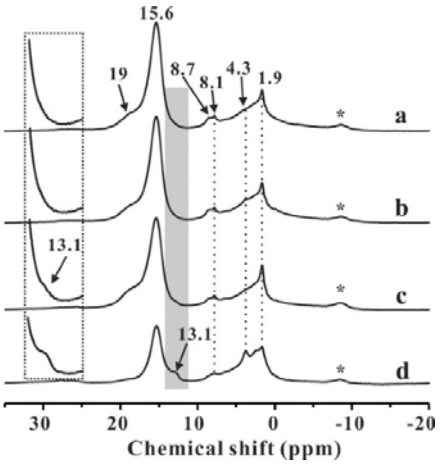

Figure R3. ^{1}H MAS NMR spectra of [D_5]pyridine adsorbed on (a) HZSM-5, (b) Ga_2O_3/ZSM-5, (c) Ga/ZSM-5(IM), and (d) Ga/ZSM-5(redox). The asterisks denote spinning sidebands.[1]

1. P. Gao, Q. Wang, J. Xu, G. Qi, C. Wang, X. Zhou, X. Zhou, N. Feng, X. Liu, F. Deng, ACS Catal. 2018, 8, 69-74.

(5) It is somewhat unfair to compare Ga(IM)/HZSM-5 and Ga(IE)/HZSM-5, when two catalysts are greatly different in the loading of Ga. In addition, Ga(IM)/HZSM-5 should also have considerable ion-

exchanged Ga species.

Response: Thanks for your suggestion. We have supplemented the comparative test of Ga(IM-D)HZSM-5 (Figure R4), having the same Ga loading as Ga(IE)HZSM-5. Their differences in HCHO and aromatics yields under low pressure condition could be ascribed to the different Ga species. The dominant species is Ga_2O_3 for Ga(IM-D)HZSM-5, and GaO^+ for Ga(IE)HZSM-5. Thus, Ga(IM-D)HZSM-5 still sightly promoted aromatics formation via HCHO-induced aromatics formation pathway. Ga(IE)HZSM-5 exhibited the opposite behavior compared with Ga(IM)HZSM-5 in low pressure condition, because cationic GaO^+ had no activity to generate HCHO.

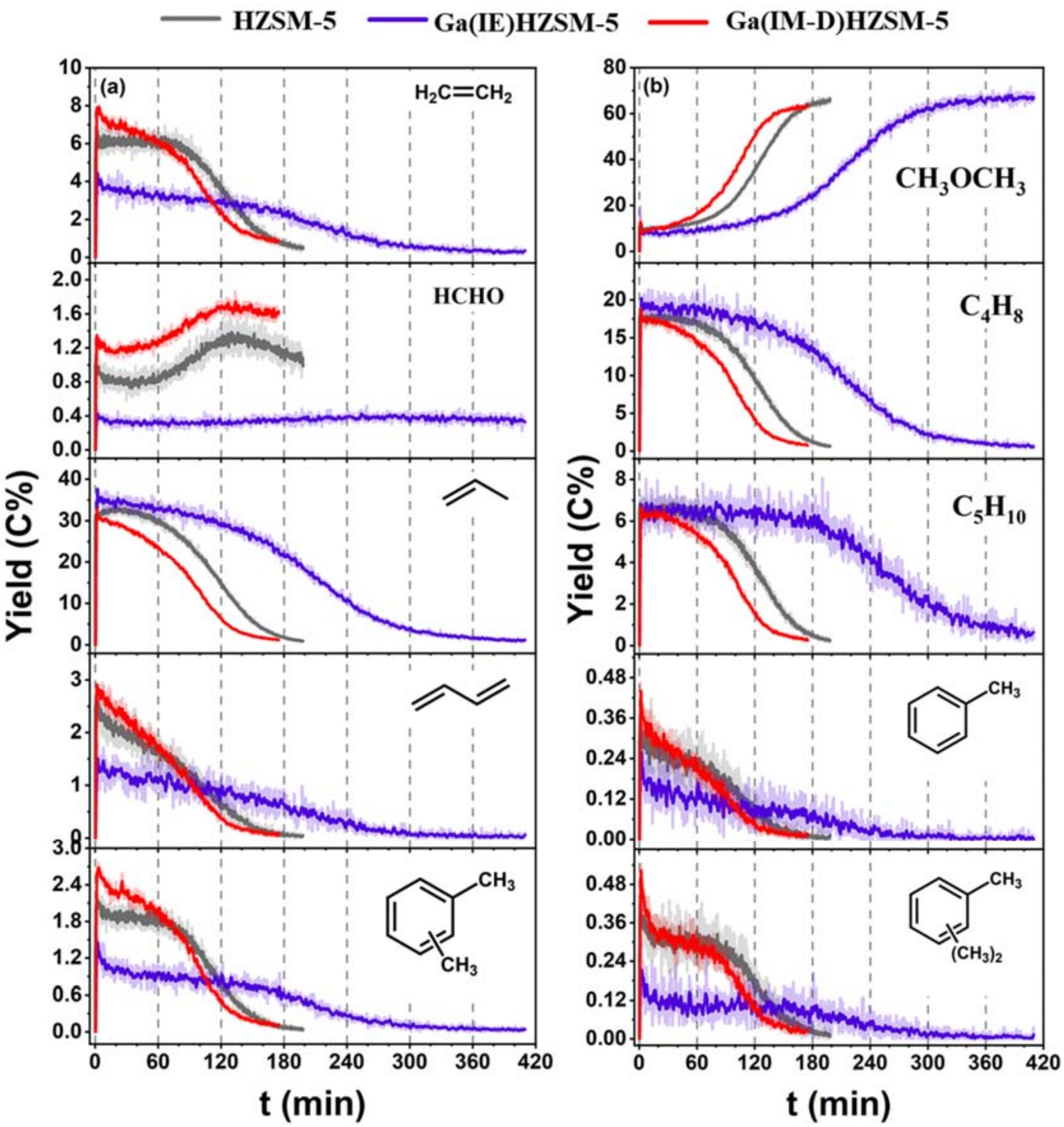

Figure R4. The real-time yields (C%) of (a) C_2H_4, HCHO, propylene, 1,3-butadiene, C8 aromatics, and (b) CH_3OCH_3, C_4H_8, C_5H_{10}, C_7H_8, C_9H_{12} in the MTH reaction over parent HZSM-5, Ga(IE)HZSM-5 and Ga(IM-D)HZSM-5. Reaction conditions: 400 °C; methanol WHSV = 12.52 $g_{MeOH}g_{catalyst}^{-1}h^{-1}$; P = 2 Torr; and each reaction proceeded

until the ethylene yield dropped to 0.5 C%. The original yield curves (transparent solid lines) obtained in the experiments have been smoothed as bright solid lines. CH_3OCH_3 = dimethyl ether; C_4H_8 = $C_4^=$ olefins; C_5H_{10} = $C_5^=$ olefins; C_7H_8 = toluene; and C_9H_{12} = C_9 aromatics.

(6) Is it possible that certain Ga is incorporated into the framework of MFI, especially after rigorous calcination treatments?

Response: Thanks for your question. In fact, we cannot absolutely deny the possibility that certain Ga may be incorporated into the framework of MFI. Nevertheless, we believe that the content of Ga in framework is very low even negligible. Several groups have reported that the ^{27}Al and ^{29}Si NMR spectra were almost unchanged after introducing Ga by impregnation (Figure R5), as well as following the H_2-O_2 treatments, suggesting that the incorporation of Ga into the framework of zeolite unlikely occurred.[1-2] However, it is also an interesting subject to study the effect of framework Ga on the methanol conversion to hydrocarbons. The incorporation of Ga into the framework of MFI would be controllable via direct synthesis.

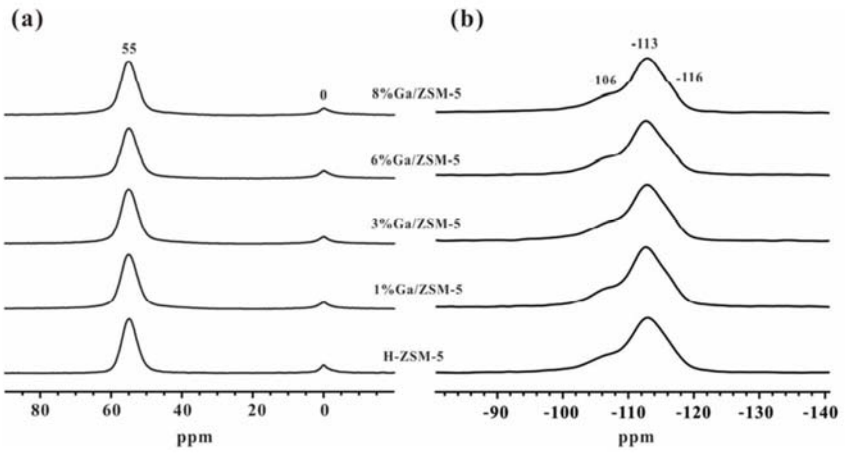

Figure R5. ^{27}Al (a) and ^{29}Si (b) MAS NMR spectra of H-ZSM-5 and Ga/ZSM-5 zeolites with different Ga loadings.[1]

1. P. Gao, J. Xu, G. Qi, C. Wang, Q. Wang, Y. Zhao, Y. Zhang, N. Feng, X. Zhao, J. Li, F. Deng, ACS Catal. 2018, 8, 9809-9820.
2. I. Nowak, J. Quartararo, E. G. Derouane, J. C. Vedrine, Appl. Catal. A-Gen. 2003, 251, 107-120.

(7) Some grammatical issues like "the usage amounts of HZSM-5, Ga(IM)/HZSM-5, single Ga_2O_3 and Ga(IM)HZSM-5(redox) were 150 mg respectively"; etc.

Response: Thanks for your correction. We have revised this sentence in the manuscript. In the catalytic testing, 150 mg of samples for HZSM-5, Ga(IM)HZSM-5, single Ga_2O_3, Ga(IM)HZSM-5(redox), Silicalite-1 and Ga(IM)Silicalite-1 were used. We have also checked out other grammatical issues and corrected them.

Reviewer #2:

The manuscript "New Insights into the Role of Formaldehyde Generated over Ga Species in Promoting Aromatics in Methanol Conversion" describes a piece of research to unravel the role gallium modified ZSM-5 in the MTG reaction. Utilising state of the art SR-PIMS analysis combined with separate online catalytic tests, the authors have shown that Ga_2O_3 species are responsible for the production of formaldehye, through the direct dehydrogenation of methanol, which results in elevated aromatics production. The findings will be of great interest to the very large MTG community, to the wider catalysis community, and also of interest to those working in the area of valorisation of feedstocks such as coal, gas and biomass.

My recommendation, based on the specific comments below, is:
Invite the authors to revise their manuscript to address specific concerns before a final decision is reached.

Scientifc comments:
The following scientific points are the main reason to ask for a revised manuscript to be submitted. The data produced by the SR-PIMS technique is very powerful, however the characterisation data of the materials made is not well described, and the experimental detail insufficient to be repeated by others.

1. The TEM images (Fig S7) are said to show that IE material does not contain Ga_2O_3 clusters, while the IM material has uniformly distributed clusters. This is not supported by the TEM images as it is only possible to discern particles around the very edge of the crystallites due to the poor TEM resolution (it looks as though the body of the crystallites are too thick for the e-beam to penetrate the material).

Response: We appreciate your question. The zeolite sizes are around 1-2 μm, so that the body of the crystallites are too thick for the e-beam to penetrate the material. It is limited by the mass thickness contrast of the material. The TEM images cannot represent the dispersion of Ga_2O_3 clusters over the whole zeolite crystallites. While, Ga_2O_3 clusters presented at the edge of the crystallites can also provide the evident morphology features. The Ga_2O_3 particles displayed relatively uniform size distributions. Moreover, combined with the XRD patterns, only the MFI framework of zeolite is identified and no bulk Ga_2O_3 phase can be detected for all the Ga-modified samples. It indicates that Ga_2O_3 clusters present in small sizes, beyond the detection limit of XRD. Thus, we revised the statement in the manuscript as follow: "Nanoscale Ga_2O_3 nanoparticles with uniform size distributions were also observed over HZSM-5 matrix as shown in TEM images (Supplementary Fig. 9)."

2. The lack of detail around band deconvolution for NH_3 TPD is concerning given that it is used to characterise the different species present.

Response: Thanks for your suggestion. We have deconvolved the NH_3-TPD curves into three main peaks (Figure R6), which located at 125, 223 and 450 °C, respectively. They correspond to the weak, medium and strong acid sites with increasing temperature. Generally, the peak at 450 °C, i.e., strong

acid sites, can be assigned to Brønsted acid sites and strong Lewis acid sites. It significantly decreased after introducing Ga due to ion exchange between Ga species and BAS. The two peaks at lower temperature mainly belong to the weak and medium Lewis acid sites.

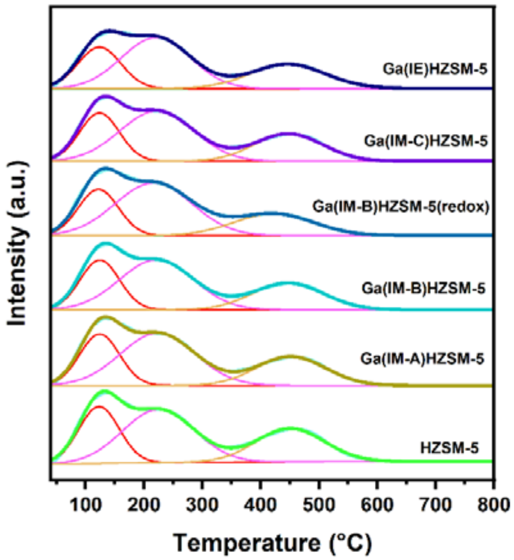

Figure R6. NH_3-TPD patterns of parent HZSM-5 and Ga-modified HZSM-5.

3. The authors offer no suggestion to the other deconvoluted bands in the FTIR spectrum at approx 3400 and 3500 cm^{-1}. It is also surprising that there are no terminal silanol species at approximately 3700 cm^{-1}. Can the authors explain why this band is absent?

Response: Thanks for your question. The FTIR spectrum of various catalysts were performed using the standard KBr pellet method. Although both the zeolites and KBr have been heated to remove water before test, the ex situ FTIR experiments inevitably affected by water adsorption. The signal at approximately 3400 and 3500 cm^{-1} could be assigned as the O-H stretching vibration of adsorbed water (Figure R7), accompanied by the O-H bending vibration at 1630 cm^{-1}. The terminal silanol species were located at approximately 3740 cm^{-1}. In our experiments, the signal of terminal silanol species was very weak. The low intensity for Si-OH may be ascribed to the effect of adsorbed water. The absence of terminal silanol signal in ex situ FTIR spectrum (KBr pellet method) was also observed by other researchers (Figure R8).

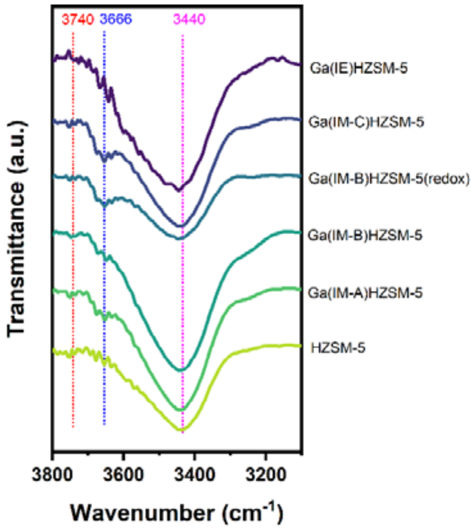

Figure R7. The FT-IR spectra of parent HZSM-5 and Ga-modified HZSM-5.

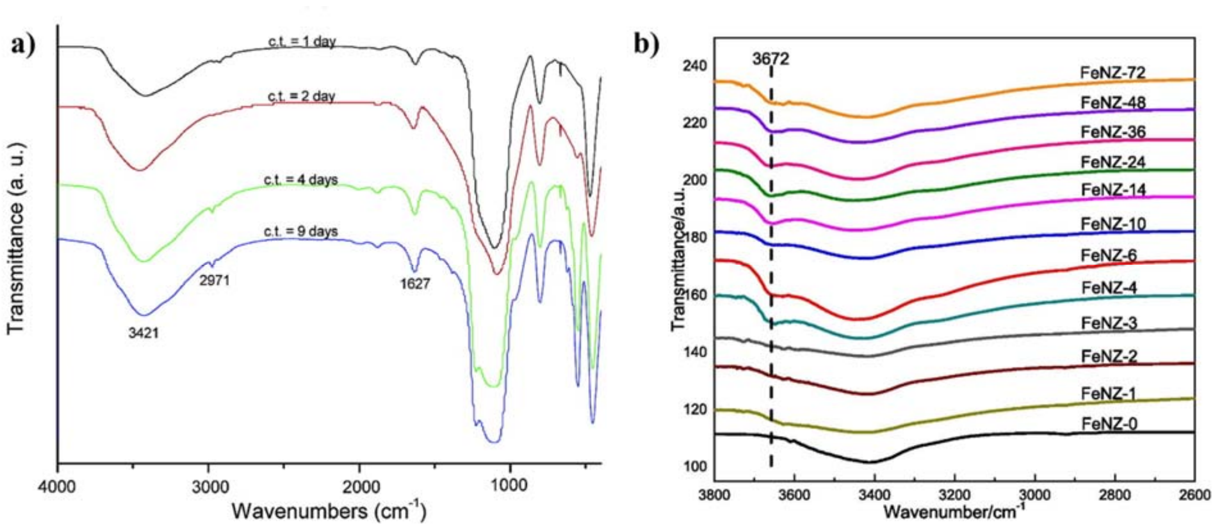

Figure R8. The FT-IR spectra of a) ZSM-5 zeolites and b) nanocrystalline H[Fe,Al]ZSM-5 zeolites with different crystallization times.[1-2]

Missing experimental detail (not exhaustive):

1) Where did the Ga and Y salts originate? Where appropriate, what grade of material was used? (e.g. methanol, Ga and Y sources, "pure hydrogen", air (synthetic or just from the lab?)?

Response: Thanks for your questions. We have added the source and purity of the reagents and gases

in the section 1.1 of the supplementary information.

2) Missing experimental details. What ramp rate was used for all heating protocols? How was the solid dissolved for ICP analysis? How were the TEM samples prepared? What were the degas conditions for BET analysis. Over what p/p0 range was applied for BET analysis? What method of FTIR was employed - transmission, DRIFTS, ATR? Were thermal treatments applied to the zeolite/KBr tablet prior to analysis? During pyridine desorption at elevated T, what duration was each T held at? What extinction coefficient was applied for LAS and BAS sites for quantitative determination? What is the uncertainty in the quantitative determination of BAS and LAS sites by pyridine adsorption? Were the catalysts tested as powders or pressed and sieved to a certain particle fraction?

Response: Thanks for your questions. 1) We have added all the heating rate in the section of methods. 2) In a typical test, 50 mg of zeolite sample was dissolved in the mixture of 2 mL of $HClO_4$, 8 mL of HF and 4 mL of HNO_3, and then diluted to 250 mL with deionized water. 3) Before TEM test, the grinded zeolite samples were dispersed in ethanol with ultrasonic treatment, and then the resulted suspension were dripped carefully on the copper grids with air drying. 4) Before nitrogen adsorption/desorption measurement, the samples were degassed at 300 °C for 30 min under vacuum conditions. 5) The P/P0 range applied for BET analysis was 0.08-0.30. 6) We employed the transmission FTIR method. 7) The zeolite/KBr tablet was heated at 105 °C for 12 h to remove water prior to analysis. 8) Keep at the desorption temperature for 30 minutes before the FTIR scan. 9) The integrated molar extinction coefficients (IMEC, cm/μmol) of BAS and LAS were obtained from previous studies.[1] *IMEC(B)*=1.67 cm/μmol and *IMEC(L)*= 2.22 cm/μmol. 10) According to the repeated experiments of pyridine adsorption of HZSM-5, we calculated that the relative deviation in the quantitative determination of BAS and LAS sites was about 5%. 11) All kinds of powder samples were pelletized, crushed and sieved to 30-40 mesh before catalytic testing.

1. C. A. Emeis, J. Catal. 1993, 141, 347-354.

3) Please could the authors add the Ga/Al ratios of the materials to Table 1.

Response: Thanks for your suggestion. We have added the Ga/Al ratios of the catalysts to Table 1.

Sample	Ga loading[a] (wt%)	Ga/Al ratio[a]	Si/Ga ratio[b]	Relative crystallinity[c] (%)	S_{BET} (m^2g^{-1})	V_{micro} (cm^3g^{-1})	Acid amount[d] (μmol g^{-1})			Total	Acid sites[e] (μmol g^{-1})		
							weak	medium	strong		BAS	LAS	B/L
HZSM-5	0	-	-	100.0	305	0.11	273.1	196.2	168.2	637.5	334.9	86.4	3.9
Ga(IM-A)HZSM-5	0.98	0.6	11.8	99.0	289	0.11	260.5	193.8	154.5	608.8	308.8	90.7	3.4
Ga(IM-B)HZSM-5	2.4	1.4	6.8	99.6	272	0.11	250.1	182.5	143.2	575.8	270.6	105.5	2.6
Ga(IM-B)HZSM-5(redox)	2.5	1.4	26.1	98.8	271	0.1	262.6	199.8	120.4	582.9	100.9	162.7	0.6
Ga(IM-C)HZSM-5	4.6	2.7	5.6	95.5	265	0.1	247.8	183.7	143.3	574.8	266.2	110.1	2.4
Ga(IE)HZSM-5	0.2	0.1	90.2	104.0	264	0.1	232.4	180.8	139.6	552.8	183.0	168.4	0.8

[a] Determined by ICP-AES. [b] Measured by XPS. [c] Calculated from XRD. [d] Measured by NH_3-TPD. [e] Measured by TF-IR of pyridine adsorption.

General comments:

1. The text needs careful revision to ensure that the scientific message is clear and can be understood by readers. I had to read the manuscript several times in order to understand the message. (e.g. sentence over lines 183-186, sentences over 227-231).

Response: Thanks for your suggestions. We have revised these sentences to make it clear.

2. The authors use language which is overly confident in my opinion: (144-146: "the lack of GaOH peak in FT-IR spectra and Ga_2O_3 particles in TEM for Ga(IE)HZSM-5 demonstrated that only cationic GaO+ was left after" - there could be other explanation's, e.g. below limit of detection; 178-180 "In other word, Ga_2O_3 could be uncontroversially assigned as the active phase in Ga(IM)HZSM-5 for the dehydrogenation of MeOH to HCHO" - "strongly indicates" would be better than "uncontroversially"). It would help readers if a line summing the aromatics product yield was added to Table S2.

Response: Thanks for your suggestions. We have revised these overly absolute expressions in the manuscript as follows. 144-146: In contrast, the lack of GaOH peak in FT-IR spectra and Ga_2O_3 particles in TEM for Ga(IE)HZSM-5 demonstrated that cationic GaO^+ was predominant after washing with plenty of deionized water. 178-180: In other words, this result strongly indicates that Ga_2O_3 should be assigned as the active phase in Ga(IM)HZSM-5 for the dehydrogenation of MeOH to HCHO. In addition, we have added the summation of aromatics yield in Table S2.

3. Line 206-208 should make reference to Table 1.

Response: Thanks for your suggestion. We have made reference to Table 1 in this section.

4. The manuscript line 319 states that the HayeSep column was used for inorganic analysis - surely this was a typographical error? CO_2 is not an inorganic molecule.

Response: Thanks for your question. We have checked this part carefully. There is no typographical error. CO_2 is indeed an inorganic molecule. It is a common method to analyze inorganic products (including CO_2) with a TCD detector connecting a packed column and organic products with a FID detector connecting a capillary column, respectively.[1-2]

1. P. Gao, J. Xu, G. Qi, C. Wang, Q. Wang, Y. Zhao, Y. Zhang, N. Feng, X. Zhao, J. Li, F. Deng, ACS Catal. 2018, 8, 9809-9820.
2. Y. Ni, W. Zhu, Z. Liu, ACS Catal. 2019, 9, 11398-11403.

Reviewer #3:

In this contribution, Yang Pan and co-workers use synchrotron-based photo-ionisation mass spectrometry to study the formaldehyde-induced conversion of methanol to aromatics (and other products) over H-ZSM-5 catalysts, either as such, or modified/ mixed with Ga^{3+}/Ga_2O_3 and potentially Y_2O_3. The study is a follow-up of a prior contribution from the same group, where they studies ZSM-

5 with and without addition of Y_2O_3 by using the same method.
In this contribution, the authors observe that aromatics formation is correlated with H_2 formation, and conclude that CH_2O formation may proceed over two different Ga sites: Ga ion-exchanged onto the Brønsted acid site of the zeolite, and Ga_2O_3 inside or outside the ZSM-5 crystals.
The study is novel and reports interesting observations that will most certainly be appreciated by the catalysis community. I suggest acceptance after properly taking into account the following comments:

1. The correlation of formaldehyde formation to hydrogen formation is based on their concomitant increase with different material combination, and I appreciate that. However, there is no direct comparison between the molar amount of H_2 and HCHO/ HC products formed in the experiments. This information should be provided in the revised manuscript. In our own research, we found that the amount of H_2 formed in similar (yet less advanced) experiments was always lower than expected, and we have not sorted out why. In case the authors made a similar observation, a discussion of this issue would be appreciated.

Response: Thanks for your suggestions. We have displayed the comparison between the molar amount of H_2 and HCHO/aromatics/hydrocarbons products in Figure R9. However, we must stress that H_2 and HCHO can be only detected by gas chromatography and mass spectrometry, respectively. The comparison for H_2 and HCHO did not take under the same conditions. The molar concentrations of H_2, C_2H_4, total aromatics and total hydrocarbons were based on the atmospheric experiments (Table S2), while that of HCHO was based on the low-pressure SR-PIMS experiments (Table S1). It showed that H_2, HCHO, C_2H_4 and total aromatics gave well linear relation with Ga content. The total hydrocarbons remain almost unchanged. Whereas we regret that the amount of H_2 formed in experiments was hard to predict owing to several routes for H_2 production. In our work, we believe that primary H_2 production comes from the direct dehydrogenation of methanol, and the dehydrogenation of alkenes and alkanes to higher unsaturated compounds accounts for a part of H_2 source. The reviewer stated that the amount of H_2 formed in their experiments was always lower than expected. We feel sorry that we don't have enough evidence to explain this question. Whether the reviewer over-estimated the amount of H_2 production. In some cases, hydrogen atom may be eliminated as water, instead of H_2.

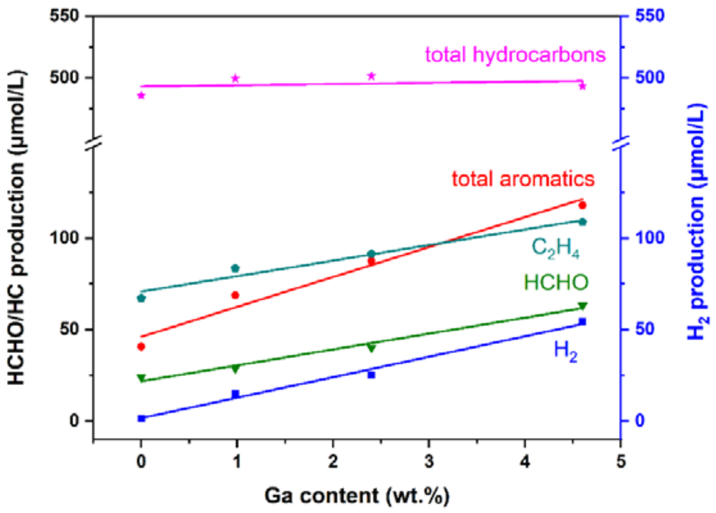

Figure R9. The mole concentrations of HCHO, C_2H_4, total aromatics, total hydrocarbons and H_2 versus Ga content. Total aromatics: the summation of C6-C9 aromatics in Table S2. Total hydrocarbon: the summation of all hydrocarbon products presented in Table S2. The mole concentrations of H_2 and C_2H_4 were calculated from their productions presented in Table S2. The mole concentration of HCHO was calculated from the yields of HCHO presented in Table S1. The concentrations of HCHO, C_2H_4, total aromatics and total hydrocarbons were calculated as the carbon atoms concentrations in corresponding products.

2. As shown by this study and the authors' prior study, as well as literature studies, formaldehyde is a precursor to aromatics formation. Moreover, according to the latest mechanistic studies of the initiation reaction, i.e. the formation of the first C-C bond in MTH, formaldehyde could either react directly with methanol/methoxy, or split to CO and H_2 followed by carbonylation of a methoxy species, to form the first C-C bond. In light of those literature studies, it is of interest to know what happens to the initiation rate when adding Ga and when adding Ga + Y_2O_3, i.e. to what extent does the formaldehyde formation affect the rate of formation of the first HC species? Please report and comment.

Response: Thanks for your question. In fact, we also have huge interest in the induction period and the first C-C bond formation in MTH. The initial products curves during the first 70 seconds over HZSM-5, Ga(IM-A)HZSM-5 and Ga(IM-A)HZSM-5/Y_2O_3 were presented in Figure R10. The interval between each point is 5 seconds, relatively high time resolution. It showed that the yields of HCHO, C_2H_4 and C_8H_{10} increased at the beginning when adding Ga, and they decreased when adding Ga+Y_2O_3, just like in steady period. Propene is the predominant hydrocarbon product in the initial period. However, the formation rate of propene at the beginning had little difference for the three catalytic experiments. In this work, the effect of formaldehyde formation on the initiation reaction is hard to confirm. The main difficulty lies in the very fast reaction processes in the induction period of MTH, especially under high temperature condition. The 5s interval is still too long to detect the first HC species. This question would be our next research point.

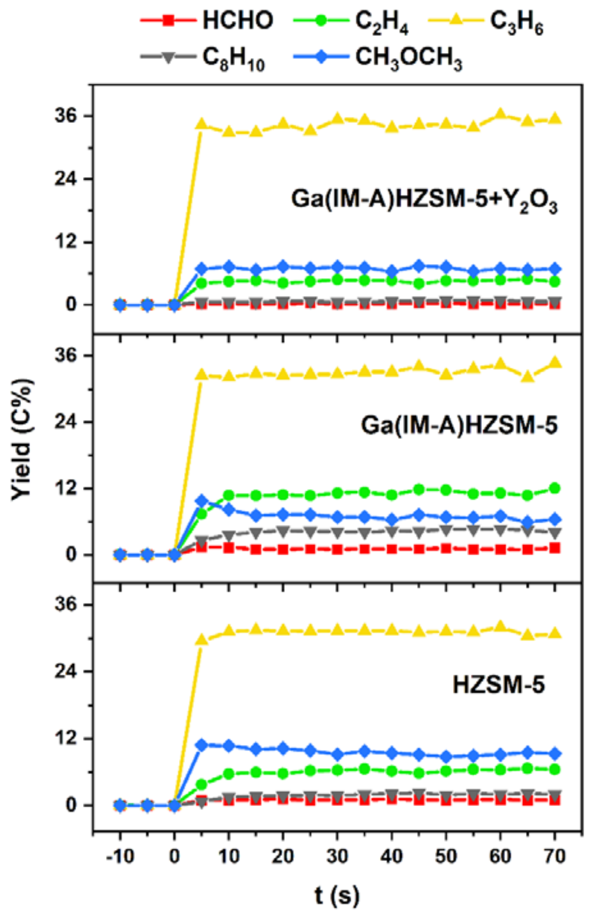

Figure R10. The initial period of MTH reaction over HZSM-5, Ga(IM-A)HZSM-5 and Ga(IM-A)H ZSM-5/Y_2O_3.

3. On page 5, the authors state: "Due to the formation of ethylene is closely associated with the aromatics-based cycle…, the yield of ethylene increased obviously with the enhanced production of aromatics." This statement may be correct, but I find it counterintuitive to correlate ethylene formation only to the aromatics cycle as long as ethylene appears before the aromatic product in Figure 3 e. Are there other aromatic products formed before ethylene, which led to the authors' statement? Did the time before appearance of each product change between materials? Please comment.

Response: Thanks for your questions. It is well accepted that the enhanced aromatics production promotes the formation of ethylene. As for ethylene appearing before the aromatic product, there exists the issue of diffusion. Smaller molecule diffuses faster than the bigger one in zeolite channel. In fact, ethylene did not appear earlier that aromatic from the Figure 3e. The yields of aromatics at the beginning were very low, but not none. In addition, we found that the appearance time of each product may change over different materials. For example, propene is the predominant hydrocarbon during the first 5 s for HZSM-5 and Ga(IM-A)HZSM-5, as shown in the response of question 2. However, all the hydrocarbon products were significantly suppressed at the beginning over Ga(IM-B)HZSM-5(redox), with much higher HCHO formation at the first 5 s. The delayed effect on hydrocarbons production may be explained by the much lower Brønsted acid sites of Ga(IM-B)HZSM-5(redox). Another

possible reason is that the high concentration HCHO causes the competitive adsorption with methanol and DME.

4. For the readers' information, please add CH_4 to Fig. 3e.

Response: Thanks for your suggestion. We have added CH_4 to Fig. 3e.

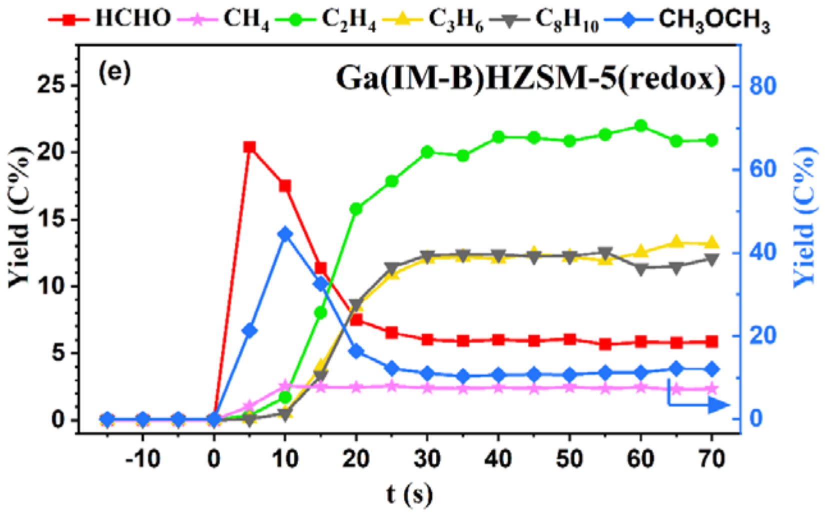

Figure R11. An enlarged view of the first 70 seconds in MTH over Ga(IM-B)HZSM-5(redox).

5. Reproducibility. To what extent have the reported results and trends between materials been confirmed by reproducibility tests? Please report and add reproducibility results with error bars to the manuscript.

Response: Thanks for your question. We have repeated the typical HZSM-5 experiments for three times to confirm the reproducibility. It showed well repeatability by SR-PIMS experiment, as shown in Figure R12.

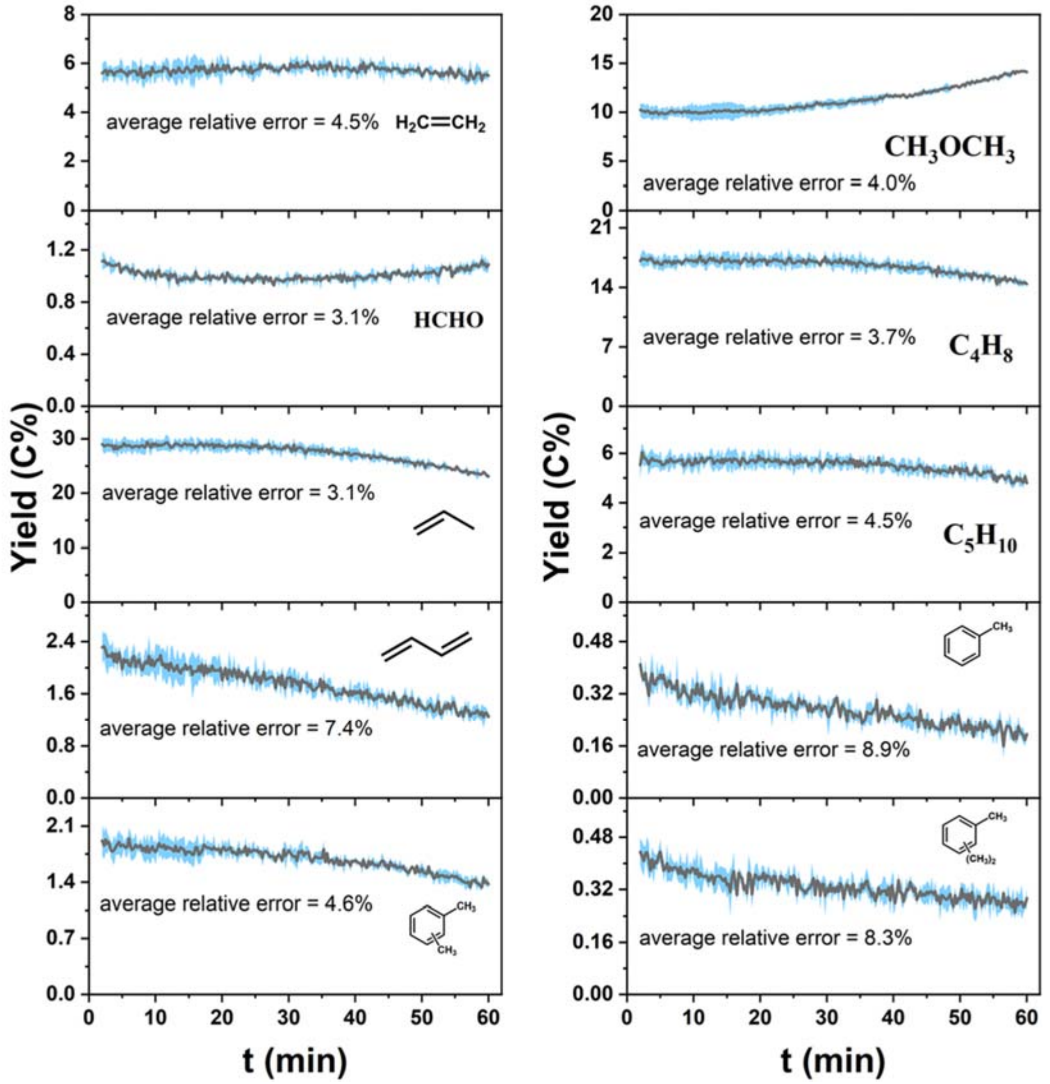

Figure R12. The reproducibility tests over parent HZSM-5 for three times. The dark gray lines represent the average value of three repeated experiments. The light blue shadows represent the error band. Reaction conditions: 400 °C; methanol WHSV = 12.52 $g_{MeOH}g_{catalyst}^{-1}h^{-1}$; P = 2 Torr; 1 h. CH_3OCH_3 = dimethyl ether; C_4H_8 = $C_4^{=}$ olefins; C_5H_{10} = $C_5^{=}$ olefins; C_7H_8 = toluene; and C_9H_{12} = C_9 aromatics.

6. Scheme 1 is too schematic. Please draw, either in main manuscript or in SI, the suggested mechanism of formation of each product, including a distinction between the roles of BAS and LAS in each reaction.

Response: We appreciate your suggestion. We have supplemented the detailed reaction routes of HCHO-induced and LAS-induced aromatics formation pathways in SI. The roles of BAS, LAS and Ga_2O_3 were marked in each process (Figure R13).

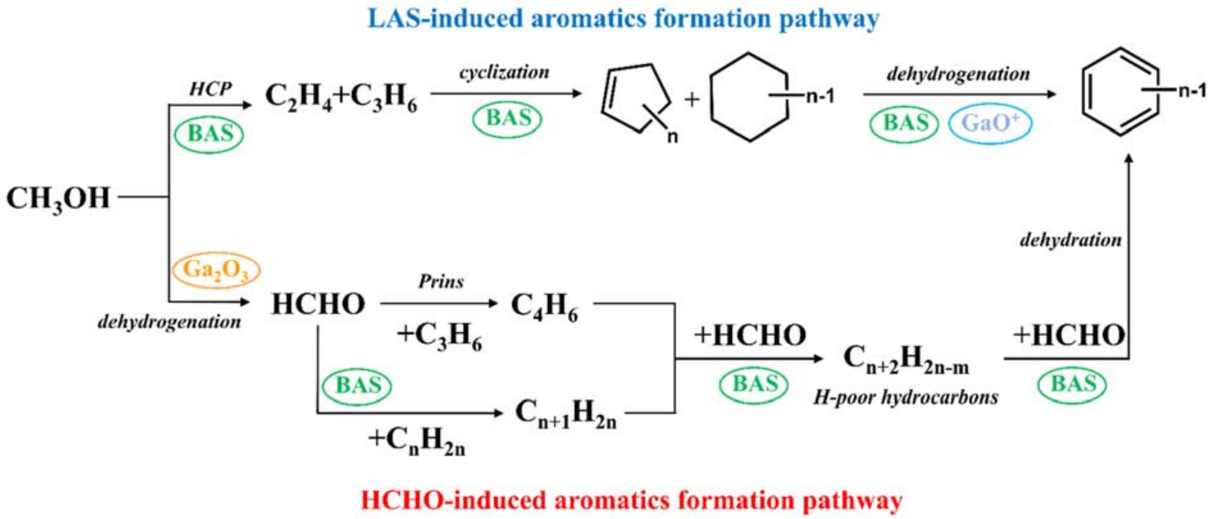

Figure R13. The details of HCHO-induced and LAS-induced aromatics formation pathway over various active centers.

7. In the prior contribution on ZSM-5 with and without Y_2O_3 addition, a direct correlation was observed between methane and formaldehyde formation over ZSM-5. Here, that is not the case. It would be interesting to know what the authors think of this difference – is the CH_4-based route completely suppressed in the presence of Ga? Please elaborate.

Response: We appreciate your question. In our previous work, we proved that formaldehyde was derived from disproportionation of methanol over ZSM-5. It can be supported by the similar formation trends of HCHO and methane, as well as their close correlation in yields. Y_2O_3 addition had only effect on eliminating HCHO, while the formation and conversion of CH_4 unchanged. In this work, we found that the presence of Ga had little effect on the formation of CH_4 (Figure 2a). Therefore, we considered that the CH_4-based route was unaffected in the presence of Ga. However, the yield of HCHO significantly increased with the increasing of Ga loading. After verification, the increment of HCHO could be attributed to the direct dehydrogenation of methanol. CH_4 is mainly originated from the disproportionation of methanol, while HCHO comes from three routes (disproportionation of MeOH (Rxn 1), hydrogen transfer from MeOH to olefins (Rxn 2) and dehydrogenation of MeOH (Rxn 3)). The correlation between methane and formaldehyde do not setup under this case.

Minor comment:

8. The manuscript needs some language improvement before publication. Much of it is well written, but then there are sentences / statements that need correction, such as the following examples:
In Abstract, line 2: "However, the active sites … are still debating."
Page 3, 6th last line: "It was suggested that there may exist some certain active sites…"
Page 3, 3rd last line: "…, which has aroused wide interest from more and more investigators recently."
Page 6, 4th last line: "… Rxn 1 and Rxn 2 could not interpret the increment of HCHO."

Response: We appreciate your suggestions. We have corrected these sentences as follows:

1. However, there are still disputes about the exact active sites and the aromatic formation

mechanisms over Ga-modified zeolites.

2. It suggested that there may exist some certain active sites located in the interface region between Ga_2O_3 and zeolite to promote MeOH into aromatics.
3. Formaldehyde (HCHO) is an important active intermediate in the MTH reaction, which has aroused wide interest from many investigators recently.
4. It suggested that the increment of HCHO did not originate from the pathway of MeOH disproportionation and hydrogen transfer.

REVIEWERS' COMMENTS:

Reviewer #1 (Remarks to the Author):

It seems that the issues raised by the reviewers in the last version have been properly responded in the revised manuscript. As a result, this reviewer suggests that the manuscript may now be acceptable for publication.

Reviewer #3 (Remarks to the Author):

The authors have provided considerate answers and made adequate changes to the revised MS and SI. I recommend acceptance of the revised contribution.
